# A Novel Topology-Based Candidate Reaction Prediction Approach for Gap-Fillings of Genome-Scale Metabolic Models

**DOI:** 10.3390/metabo16040258

**Published:** 2026-04-12

**Authors:** Jiajun Qu, Kai Wang

**Affiliations:** Key Laboratory of Advanced Process Control for Light Industry (Ministry of Education), School of Internet of Things Engineering, Jiangnan University, 1800 Lihu Road, Wuxi 214122, China; 6231905046@stu.jiangnan.edu.cn

**Keywords:** genome-scale metabolic model, model reconstruction, graph convolutional network, hypergraph learning, squeeze-and-excitation network

## Abstract

**Background**: It is significant to predict and fill metabolic reaction gaps (gap-fillings) for reconstructions of high-quality genome-scale metabolic models (GEMs). Currently, many existing optimization-based gap-filling methods have to rely on phenotypic data, while performances of topology-based approaches by deep learning algorithms need to be further improved. **Methods**: This paper proposes a novel topology-based approach (GHCN-SE) of predicting confidence scores of candidate reactions, which can be used for gap-fillings of GEMs. The topological features of GEMs are fully extracted by simultaneously using graph and hypergraph convolutional networks, such that both associations of metabolites in the same reaction and higher-order interactions of metabolites within reactions can be captured. After the feature fusion, we further employ the squeeze-and-excitation network to enhance features. **Results**: The reaction prediction and reaction recovery experiments through 5-fold cross validations on 108 high-quality BiGG GEMs show that the proposed GHCN-SE is superior to other related methods. The ablation study further demonstrates the contributions of the graph convolutional network, hypergraph convolutional network, and squeeze-and-excitation network in GHCN-SE. In addition, the visualization study interprets the effectiveness of GHCN-SE. **Conclusions**: For potential applications in metabolic engineering, biomedicine, etc., this proposed GHCN-SE can be used to further improve the phenotypic prediction accuracy of the draft GEM generated from automated reconstruction tools.

## 1. Introduction

Genome-scale metabolic models (GEMs) can reveal the association between genotype and phenotype through gene–protein-reaction (GPR) rules [1,2,3,4], which integrate all known genes, metabolites, and metabolic reactions of organisms into mathematical frameworks and are reconstructed from genome sequence data [5,6]. GEMs are widely utilized for biological phenotype predictions [7,8], metabolic engineering [9,10] and biomedicine [11], etc. In recent years, many automated reconstruction tools of GEMs have emerged, which can effectively accelerate the reconstructions and reduce experimental costs for GEMs compared with traditional manual reconstructions [12]. These automated tools such as ModelSEED [13], CarveMe [14] and Gapseq [15] generally reconstruct GEMs from genome sequence data and relevant metabolic reaction databases. However, draft GEMs reconstructed by these tools commonly include numerous network gaps, which are due to incomplete genome annotations or underground reactions [16,17]. Therefore, it is essential to identify and fill these network gaps in GEMs.

A series of optimization-based tools was proposed to achieve gap-fillings. GrowMatch [18] can resolve growth prediction inconsistencies by identifying the minimal set of restrictions that need to be imposed on the model. However, the method based on optimization algorithms usually excessively depends on phenotypic data and is difficult to apply to non-model organisms. FASTGAPFILL [19] identifies a minimum reaction set from biochemical reaction databases to restore metabolic flux in dead-end reactions. However, the method usually pursues mathematically optimal solutions and ignores biological feasibility. Meneco [20] addresses gap-filling problems by utilizing answer set programming (ASP), which can identify missing reactions and achieve gap-fillings in draft GEMs at high degradation rates. However, the method based on optimization algorithms usually tends to prioritize the shortest reaction paths and lacks biological feasibility.

With the development of deep learning, the advanced deep learning-based methods are increasingly applied to gap-fillings of GEMs [21,22]. These deep learning-based methods typically construct the network structure as graphs or hypergraphs for hyperedge predictions of missing reactions, which combine with candidate reaction pools to achieve gap-fillings. CHESHIRE [23] employs Chebyshev spectral convolutional networks to capture topological features of metabolic networks. This strategy enables the prediction of missing reactions and selects suitable candidate reactions from a universal reaction pool to achieve gap-fillings. HGNNP [24] is a hyperedges prediction strategy that employs a two-stage hypergraph convolution approach for capturing higher-order interactions between metabolites and reactions. DSHCNet [25] further constructs homogeneous and heterogeneous graphs to identify substrates and products in GEMs based on Chebyshev spectral convolutional networks and selects appropriate reactions to implement gap-fillings. Multi-HGNN [26] further combines a pre-trained model to perform feature extractions from molecular graphs of metabolites, and integrates directed graphs with hypergraphs to predict missing reactions in GEMs. However, the prediction performances of these topology-based approaches by deep learning algorithms need to be further improved.

In this study, we propose a novel topology-based approach, named GHCN-SE (Graph and Hypergraph Convolution Networks—Squeeze and Excitation), to predict and fill the candidate reactions in GEMs. GHCN-SE consists of three modules. In the feature extraction and fusion module, we utilize draft GEMs as input and simultaneously use a graph convolutional network and a hypergraph convolutional network to extract both associations of metabolites in the same reaction and higher-order interactions of metabolites within reactions, respectively, where learnable metabolite initial embeddings can be updated in the training phase. In the feature enhancement module, we employ a squeeze-and-excitation network to enhance metabolite features after the fusion of topological features. In the output module, GHCN-SE uses a multi-layer perceptron to yield confidence scores of candidate reactions. To evaluate the performances of GHCN-SE, we selected 108 high-quality BiGG GEMs from the BiGG database [27] for training and testing. We conducted 5-fold cross validations and compared the evaluation metrics with state-of-the-art deep learning-based methods by evaluating reaction prediction performances and reaction recovery performances. The reaction prediction results demonstrate that the proposed GHCN-SE has the best performance metrics compared with other related methods. Moreover, we further analyzed the results classified by the network scales and biological categories of GEMs. The reaction recovery results demonstrate that GHCN-SE can more effectively identify the reactions within metabolic networks from real candidate reactions compared with other related methods. Our ablation study shows the effectiveness of the graph convolutional network, hypergraph convolutional network, and squeeze-and-excitation network in GHCN-SE for candidate reaction predictions. Moreover, a visualization study was conducted to interpret the effectiveness of the feature extraction and enhancement.

## 2. Materials and Methods

### 2.1. Overview

**Figure 1:**
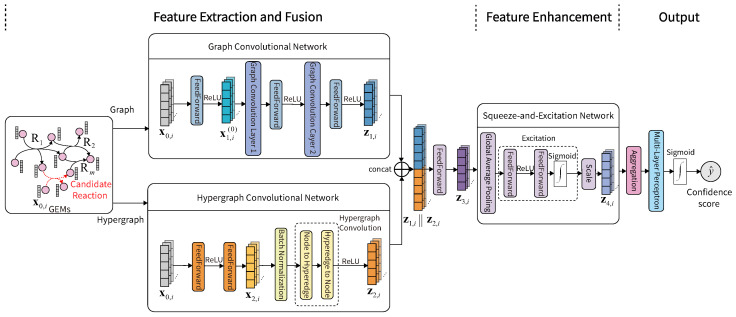
Overall architecture of the proposed GHCN-SE.

The overall architecture of the topology-based candidate reaction prediction approach for gap-fillings of GEMs, named GHCN-SE, is shown in Figure 1. GHCN-SE can output the confidence scores of candidate reactions according to the structures of draft GEMs. The confidence scores can be used to achieve gap-fillings in the draft GEMs. GHCN-SE is composed of three modules. The feature extraction and fusion module is to extract the topological features of GEMs, the feature enhancement module is to refine the features, and the output module is to aggregate features and output the confidence scores of candidate reactions.

### 2.2. Data Preprocessing

For the model training and testing of our proposed GHCN-SE approach, we chose 108 high-quality BiGG GEMs [27] referring to previous related works in [23,25,26]. The details of these GEMs can be found in Supplementary Table S3. The metabolic reactions in a GEM are regarded as the positive reaction samples of the corresponding dataset. For each positive reaction sample, we replaced a random metabolite with another random metabolite from the BiGG database, and generated a corresponding negative reaction sample. Then, we obtained the dataset with a 1:1 ratio between positive and negative samples (see Supplementary Figure S1). For 5-fold cross validations, we split the dataset for training and testing at a ratio of 4:1 with the same number of positive and negative reaction samples in each of five rounds, where the input network structures are constructed from positive reaction samples of the training set during training and testing. Any GEM can be described by a graph and a hypergraph, where all nodes, edges, and hyperedges are derived from positive samples of training set. According to CHESHIRE [23], the candidate reaction pool from the BiGG database [27] was used for reaction recovery evaluations, and it contains 10,393 metabolites and 16,337 reactions.

### 2.3. Feature Extraction and Fusion Module

In this module, we simultaneously use a graph convolutional network to extract associations of metabolites in the same reaction and a hypergraph convolutional network to extract the higher-order interactions of metabolites within reactions, and the extracted features are further fused.

We construct a graph G=(V,E) using the topology of the GEM. We represent each reaction as a fully connected subgraph. All metabolites are treated as nodes, and undirected edges are established between every pair of metabolites involved in the same reaction. The reversible reactions are treated as unidirectional reactions and compartmentalized metabolites are treated as distinct nodes. We denote the set of metabolites as V and denote the set of edges as E in the positive training subset. The edges can capture associations of metabolites in the same reaction of the positive training subset. Let $A\in {\left\{0,1\right\}}^{n\times n}$ denote the adjacency matrix of G, where $A_{ij}=1$ if metabolites *i* and *j* are involved in the same reaction, $A_{ij}=0$ otherwise. An illustrative example of constructing the graph and adjacency matrix for a GEM can be found in Figure S6A. $D=\mathrm{diag}\left(d_{1},d_{2},\ldots ,d_{n}\right)\in R^{n\times n}$ is a degree matrix with $d_{i}=\sum_{j=1}^{n}A_{ij}$. Simultaneously, we construct a hypergraph H=(V,R) using the network topology of the GEM. All compartmentalized metabolites are treated as distinct nodes. We denote the set of metabolites as V and denote the set of *m* hyperedges as R in the positive training subset. Each hyperedge represents a metabolic reaction in the positive training subset. $\Delta_{v}\in R^{n\times n}$ is the degree diagonal matrix of nodes, $\Delta_{r}\in R^{m\times m}$ is the degree diagonal matrix of hyperedges, *m* denotes the number of reactions, $H\in R^{n\times m}$ denotes the hypergraph incidence matrix, $H_{ij}=1$ denotes that node $v_{i}$ is contained within hyperedge $e_{j}$, $H_{ij}=0$ otherwise, *n* denotes the number of metabolite nodes, and *m* represents the number of reaction hyperedges. An illustrative example of constructing the hypergraph and incidence matrix for a GEM can be found in Figure S6B. We represent the metabolite initial embeddings as $x_{0,i}\in R^{N_{0}}$ where i=1,2,…,n and *n* is the number of metabolite nodes in training reaction samples and the candidate reactions. At the beginning, we randomly initialize these initial embeddings through a standard normal distribution and continuously update these initial embeddings during the training.

The graph convolutional network [28] can extract associations of metabolites in the same reaction in GEM. To map the initial embeddings into a feature space suitable for the graph convolutional network, we first employ a linear adaptation projection layer:

(1) $$x_{1,i}^{\left(0\right)}=\mathrm{ReLU}\left(W_{1}x_{0,i}+b_{1}\right),$$

where i=1,2,…,n, ReLU(·) is a Rectified Linear Unit activation function, $W_{1}\in R^{N_{1}\times N_{0}}$ is a learnable weight matrix, and $b_{1}\in R^{N_{1}}$ is a learnable bias vector. We define $A_{1}\in R^{n\times n}$ as

(2) $$A_{1}=D^{-1}\left(A+I\right),$$

where $I\in R^{n\times n}$ denotes an identity matrix. We employ a two-layer graph convolutional network, which satisfies

(3) $$x_{1,i}^{\left(k+1\right)}=\mathrm{ReLU}\left({\left(W_{2}^{\left(k\right)}X_{1}^{\left(k\right)}A_{1}\right)}_{:,i}+b_{2}^{\left(k\right)}\right),$$

where i=1,2,…,n, k=0,1, $X_{1}^{\left(k+1\right)}=\left[x_{1,1}^{\left(k+1\right)}\;x_{1,2}^{\left(k+1\right)}\;\cdots \;x_{1,n}^{\left(k+1\right)}\right]$, ${\left(\cdot \right)}_{:,i}$ denotes the *i*-th column of matrix ·, $W_{2}^{\left(0\right)}\in R^{N_{2}\times N_{1}}$ and $W_{2}^{\left(1\right)}\in R^{N_{2}\times N_{2}}$ are learnable parameter matrices, and $b_{2}^{\left(k\right)}\in R^{N_{2}}$ are learnable bias vectors. The two-layer graph convolutional network gives extracted feature representations of the graph G as $z_{1,i}=x_{1,i}^{\left(2\right)}\in R^{N_{2}}$.

Simultaneously, the hypergraph convolutional network [29] aims to extract higher-order interactions of metabolites within reactions. We feed $x_{0,i}$ into a multi-layer perceptron and apply the batch normalization:

(4) $$x_{2,i}=\mathrm{BatchNorm}\left(W_{4}\mathrm{ReLU}\left(W_{3}x_{0,i}+b_{3}\right)\right),$$

where i=1,2,…,n, $W_{3}\in R^{N_{2}\times N_{0}}$ and $W_{4}\in R^{N_{3}\times N_{2}}$ are learnable weight matrices, and $b_{3}\in R^{N_{2}}$ is a learnable bias vector. We use the hypergraph convolutional network to apply a two-stage hypergraph convolution, which satisfies

(5) $$Y=H^{T}\Delta_{v}^{-1/2}X_{2}^{T}$$

(6) $$Z_{2}=\mathrm{ReLU}\left(\Delta_{v}^{-1/2}HW_{5}\Delta_{r}^{-1}Y\right)$$

where $X_{2}=\left[x_{2,1}\;x_{2,2}\;\cdots \;x_{2,n}\right]$, $H\in R^{n\times m}$ denotes the hypergraph incidence matrix, $\Delta_{v}\in R^{n\times n}$ is the degree diagonal matrix of nodes, $\Delta_{r}\in R^{m\times m}$ is the degree diagonal matrix of hyperedges, and $W_{5}\in R^{m\times m}$ is a learnable weight matrix. The hypergraph convolutional network gives extracted feature representations of the hypergraph H as $z_{2,i}\in R^{N_{3}}$, i=1,2,…,n.

The extracted feature embeddings by the graph convolutional network and the hypergraph convolutional network are fused as

(7) $$z_{3,i}=W_{6}(z_{1,i}\left|\right|z_{2,i})+b_{6},$$

where i=1,2,…,n, $W_{6}\in R^{N_{3}\times \left(N_{2}+N_{3}\right)}$ is a learnable weight matrix, and $b_{6}\in R^{N_{3}}$ is a learnable bias vector.

### 2.4. Feature Enhancement Module

In this module, we use the squeeze-and-excitation network [30] to perform feature enhancements on the fused features. By taking the global average pooling (squeeze) operation, we can calculate the squeeze vector $s_{0}$ as

(8) $$s_{0}=\frac{1}{K_{0}}\sum_{i\in M_{k}}z_{3,i},$$

where $M_{k}$ is the set of metabolites of the candidate reaction $R_{k}$, k=1,2,…,n, and $K_{0}$ is the number of the metabolites. By taking the excitation operation, we calculate the channel-level weight vector $s_{1}\in R^{N_{3}}$ as

(9) $$s_{1}=\mathrm{sigmoid}\left(W_{8}\mathrm{ReLU}\left(W_{7}s_{0}\right)\right),$$

where sigmoid(·) is a Sigmoid activation function, $W_{7}\in R^{N_{4}\times N_{3}}$ and $W_{8}\in R^{N_{3}\times N_{4}}$ are learnable weight matrices. By taking the scale operation, we obtain the enhanced feature embeddings as

(10) $$z_{4,i}=\mathrm{LayerNorm}\left(z_{3,i}⊙s_{1}\right),$$

where $i\in M_{0}$, LayerNorm(·) is a layer normalization function, and ⊙ denotes the element-wise product of vectors.

### 2.5. Output Module

For any reaction $R_{k}$, k=1,2,…,m, we use the average pooling and a linear layer to yield the feature representations of reactions as

(11) $$r_{k}=\mathrm{ReLU}\left(W_{9}\left(\frac{1}{|M_{k}|}\sum_{i\in M_{k}}z_{4,i}\right)+b_{9}\right),$$

where $M_{k}$ is the set of metabolites of the reaction $R_{k}$, $|M_{k}|$ is the number of the metabolites, $W_{9}\in R^{N_{5}\times N_{3}}$ is a learnable weight matrix, and $b_{9}\in R^{N_{5}}$ is a learnable bias vector. Subsequently, we employ a multi-layer perceptron to yield the reaction confidence score as

(12) $${\hat{y}}_{k}=\mathrm{sigmoid}\left(W_{10}r_{k}+b_{10}\right),$$

where k=1,2,…,m, $W_{10}\in R^{1\times N_{5}}$ is a learnable weight matrix, and $b_{10}\in R^{1}$ is a learnable bias vector.

### 2.6. Model Training

In the training phase, the binary cross-entropy loss function is chosen as

(13) $$L=-\frac{1}{N_{\mathrm{batch}}}\sum_{i=1}^{N_{\mathrm{batch}}}\left(y_{i}\log{\hat{y}}_{i}+\left(1-y_{i}\right)\log\left(1-{\hat{y}}_{i}\right)\right),$$

where $i=1,2,\ldots ,N_{\mathrm{batch}}$, $N_{\mathrm{batch}}$ denotes the batch size during training, $y_{i}\in \left\{0,1\right\}$ represents the ground truth label, where 1 indicates a positive sample, 0 indicates a negative sample, and ${\hat{y}}_{i}$ denotes the confidence score predicted by the GHCN-SE (see Equations (1)–(12)). The graph and hypergraph are constructed from the positive samples subset in the training and testing. The Adam optimizer [31] can be utilized to minimize the loss function as in Equation (13) for updating the learnable parameters (see Supplementary Table S1) and initial embeddings $x_{0,i}$, i=1,2,…,n with an initial learning rate of $5\times 10^{-4}$. The hyperparameters are tuned via 5-fold cross-validations on iBWG_1329 dataset. We can evaluate the prediction performances of a set of candidate hyperparameters and select the hyperparameters with the best performances for the model trainings and testings for all the other datasets. All experiments were performed using an NVIDIA RTX 4090 GPU (24 GB memory), which supports the software environment of CUDA 12.8, Python 3.10, and PyTorch 1.13.1.

### 2.7. Gap-Filling by the Model

We utilize the draft GEM as input for GHCN-SE to train the model. In the training phase, the metabolic reactions in a draft GEM are regarded as the positive reaction samples and are used to generate the corresponding negative reaction samples. The graph and hypergraph can be constructed from the positive reaction samples. The trained GHCN-SE is employed to output confidence scores of all candidate reactions. According to CHESHIRE [23], we can use confidence scores and similarity scores of candidate reactions to select appropriate reactions for gap-fillings of draft GEMs. Then, the high-quality GEMs filled by GHCN-SE can be obtained.

### 2.8. Evaluation Metrics

We utilize five metrics to evaluate prediction performances of GHCN-SE and other related approaches, which are AUPRC, recall, F1 score, accuracy, and precision. The mathematical formulations of the above performance metrics can be expressed as

(14) $$\mathrm{Recall}=\frac{\mathrm{TP}}{\mathrm{TP}+\mathrm{FN}},$$

(15) $$F1\;\mathrm{score}=\frac{2\mathrm{TP}}{2\mathrm{TP}+\mathrm{FP}+\mathrm{FN}},$$

(16) $$\mathrm{Accuracy}=\frac{\mathrm{TP}+\mathrm{TN}}{\mathrm{TP}+\mathrm{TN}+\mathrm{FP}+\mathrm{FN}},$$

(17) $$\mathrm{Precision}=\frac{\mathrm{TP}}{\mathrm{TP}+\mathrm{FP}},$$

where TP denotes the number of true positive samples, TN denotes the number of true negative samples, FP represents the number of false positive samples, FN represents the number of false negative samples. The Area Under the Precision–Recall Curve (AUPRC) is used to evaluate performances of the model in scenarios involving dataset imbalance and uneven sample distribution. We use recovery rates to evaluate the recovery performances of GHCN-SE to recover reactions from the candidate reaction pool. The recovery rate is the ratio of positive samples to the candidate reactions with top confidence scores (top 25, top 50, top 100, and top *N*), where *N* is the number of positive samples of the testing subset in any dataset.

## 3. Results

### 3.1. Reaction Prediction Evaluation

**Figure 2:**
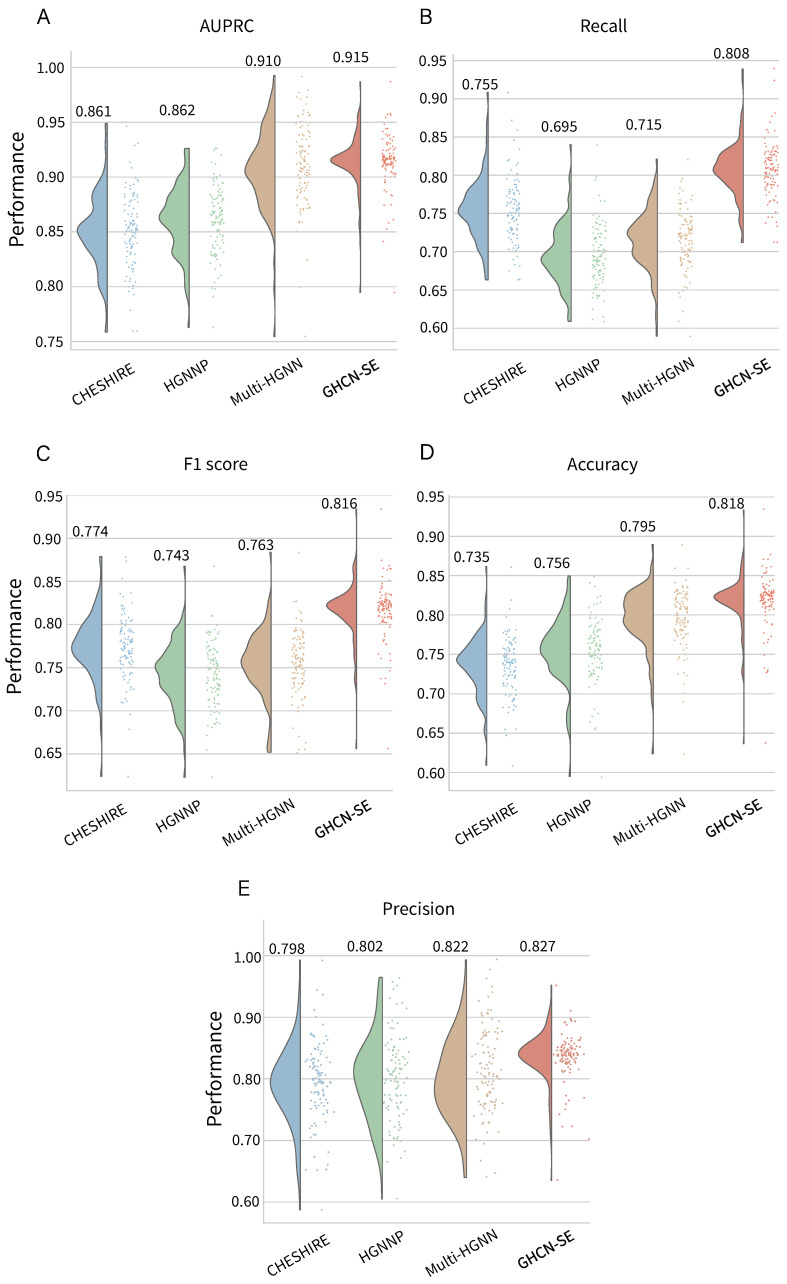
Raincloud plots and average values of reaction prediction performances on 108 BiGG GEMs for GHCN-SE and three baseline models, in terms of (**A**) AUPRC, (**B**) recall, (**C**) F1 score, (**D**) accuracy, and (**E**) precision.

To evaluate the prediction performances of our proposed GHCN-SE, we conducted performance comparisons against state-of-the-art deep learning methods. There are three different models, namely CHESHIRE [23], HGNNP [24] and Multi-HGNN [26]. We used the evaluation metrics mentioned in the evaluation metrics section for comparisons. Figure 2 shows the distributions and average values of the performance metrics of GHCN-SE on 108 high-quality BiGG GEMs. The detailed performances of GHCN-SE can be found in Supplementary Table S4. GHCN-SE achieves the best performance in terms of these five metrics compared with all the baseline methods. To be specific, GHCN-SE achieves average AUPRC, recall, F1 score, accuracy, and precision scores of 0.915, 0.808, 0.816, 0.818, and 0.827, respectively, which outperform the second-best model (CHESHIRE or Multi-HGNN) by 0.5%, 7%, 15.4%, 2.9%, and 0.6%. Moreover, GHCN-SE achieves the highest average AUPRC, recall, F1 score, accuracy, and precision on 57.4% (62/108), 98.1% (106/108), 93.5% (101/108), 82.4% (89/108), and 31.5% (34/108) of the datasets, respectively.

The results demonstrate the effectiveness of our proposed model in predicting candidate reactions for GEMs. Furthermore, the significant improvements in recall and F1 score exhibit the superiority of GHCN-SE in correctly identifying positive samples, which indicates that GHCN-SE exhibits outstanding performances in prediction candidate reactions.

**Figure 3:**
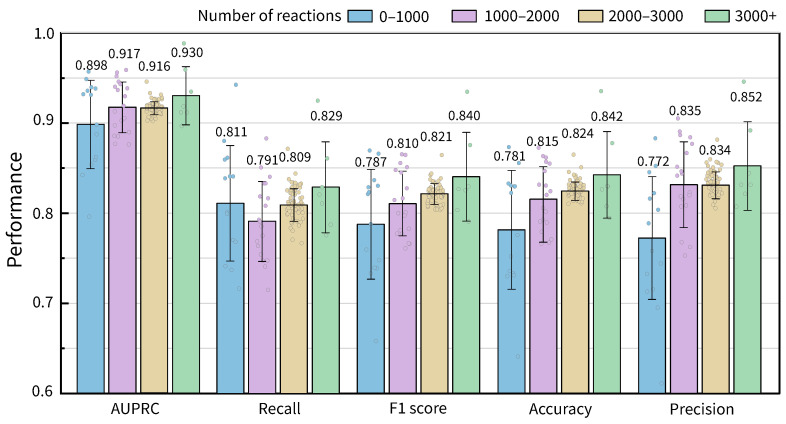
Bar chart of the average values and distributions of reaction prediction performances of GHCN-SE under different network scales, with error bars representing the standard deviation.

To discuss the influence of metabolic network scales (defined as the number of reactions) on the prediction performances of the GHCN-SE, we classified the 108 GEMs into four scale classes according to the number of reactions in GEMs. Figure 3 shows average values of performance metrics of GHCN-SE on four scale classes of the number of reactions in GEMs. The results show that overall reaction prediction performances of GHCN-SE increase with the scales of GEMs, due to the increasing number of training samples.

**Figure 4:**
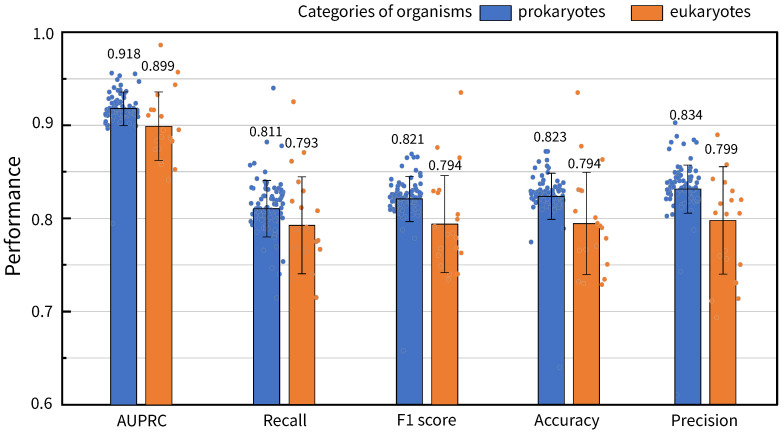
Bar chart of the average values and distributions of reaction prediction performances of GHCN-SE classified by species, with error bars representing the standard deviation.

Similarly, to verify the impact of species classifications on the prediction performances of the GHCN-SE, we discussed the reaction prediction performances of GHCN-SE for GEMs of prokaryotic organisms (*Escherichia coli* str. K-12 substr. MG1655, etc.) and eukaryotic organisms (*Homo sapiens*, etc.), where the proportions of prokaryotic and eukaryotic organisms account for 81.5% (88/108) and 18.5% (20/108) of the 108 datasets, respectively. Figure 4 presents average prediction performance values of GHCN-SE on the prokaryotic and eukaryotic GEMs among the 108 BiGG datasets. The results indicate that GHCN-SE exhibits a better performance in prokaryotic organisms than eukaryotic organisms across all evaluation metrics. The results may be attributed to the fact that metabolic networks of prokaryotes have lower complexity than metabolic networks of eukaryotes.

### 3.2. Reaction Recovery Evaluation

To further test the performances of our proposed GHCN-SE to recover reactions from the candidate reaction pool, we conducted reaction recovery evaluations on 108 high-quality BiGG GEMs. The training set was the same as the study of reaction prediction evaluations, and we replaced the negative testing samples with all the 16,337 reactions (except those already in the GEM) from the BiGG universal reaction pool in the testing phase. As introduced above, we used the recovery rate, defined as the ratio of positive samples to candidate reactions with top confidence scores (top 25, top 50, top 100, and top *N*), to measure the recovery performances, where *N* is the number of positive samples of the testing set in any of 108 GEMs.

**Figure 5:**
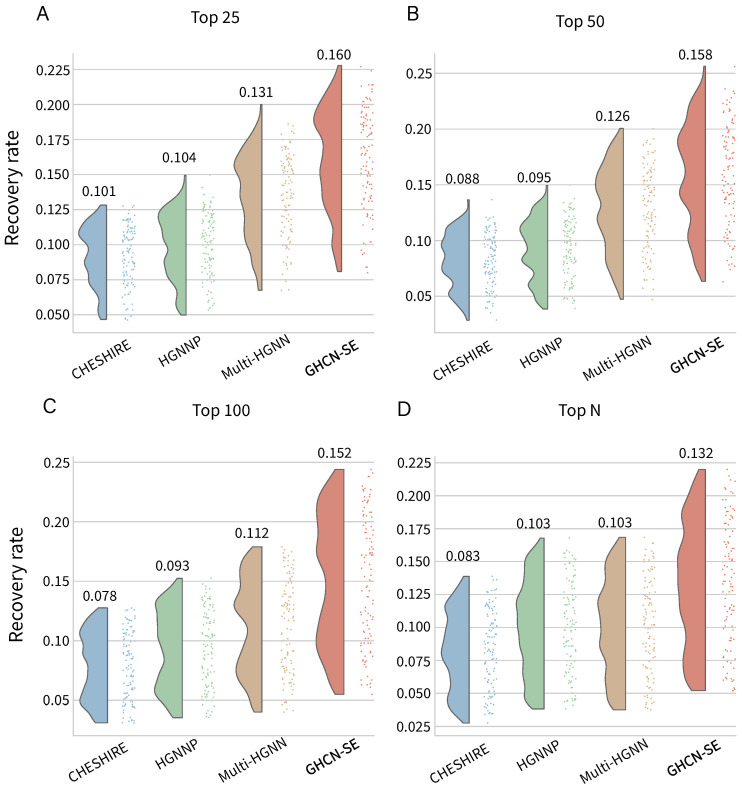
Raincloud plots and average values of reaction recovery performances on 108 BiGG GEMs for GHCN-SE and three baseline models, by selecting top 25, 50, 100, and *N* reactions, respectively. (**A**) Top 25, (**B**) Top 50, (**C**) Top 100, and (**D**) Top *N*.

Figure 5 gives the distributions and the average values of recovery rates of GHCN-SE. The detailed values of recovery rates can be found in Supplementary Table S5. We observe that GHCN-SE significantly outperforms other baseline methods. Specifically, the recovery rates for Top 25, Top 50, Top 100, and Top *N* achieve 0.160, 0.158, 0.152, and 0.132, which outperform the second-best model (Multi-HGNN) by 22.1%, 25.3%, 35.7%, and 28.2%, respectively. Moreover, GHCN-SE achieves the highest recovery rate on all of the datasets. For instance, GHCN-SE can identify the triose-phosphate/phosphate translocator (BiGG ID: TPTPt_m) in the iLB1027_lipid GEM (*Phaeodactylum tricornutum* CCAP 1055/1) within the top 25 confidence scores of the reaction recovery evaluation, and other baseline methods fail to effectively perform the gap-filling for this reaction.

The results demonstrate that the proposed GHCN-SE can better identify and fill reaction gaps based on the candidate reactions and the topology description of a given GEM.

### 3.3. Ablation Experiment

To evaluate the effectiveness of different networks within the model, we conducted ablation studies on GHCN-SE and its variant approaches in the reaction prediction evaluations with the same datasets and hyperparameters. HCN-SE, GCN-SE, and GHCN are three simplified variant approaches of GHCN-SE by respectively removing the graph convolutional network, hypergraph convolutional network and squeeze-and-excitation network.

**Figure 6:**
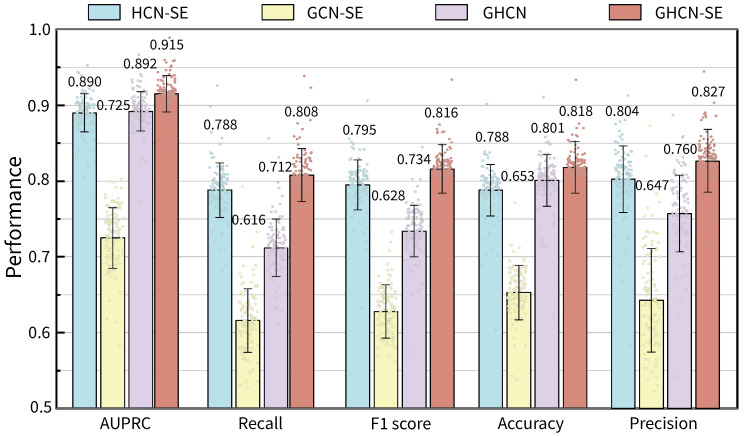
Bar chart of the average values and distributions of prediction performances of GHCN-SE and three variant approaches on 108 BiGG GEMs, with error bars representing the standard deviation.

The performance metrics of GHCN-SE and its three variant approaches on 108 high-quality BiGG GEMs are shown in Figure 6. We can observe that GHCN-SE achieves the best performances compared with the other three variant approaches in AUPRC, recall, F1 score, accuracy and precision, respectively. To be specific, compared with HCN-SE, GHCN-SE exhibits improvements of 2.8%, 2.5%, 2.6%, 3.8%, and 2.9% in AUPRC, recall, F1 score, accuracy and precision, respectively. Furthermore, GHCN-SE surpasses GCN-SE with improvements of 26.2%, 31.2%, 29.9%, 25.3%, and 27.8% in AUPRC, recall, F1 score, accuracy and precision, respectively. Moreover, GHCN-SE achieves improvements of 2.6%, 13.5%, 11.2%, 2.1%, and 8.8% compared with GHCN in AUPRC, recall, F1 score, accuracy and precision, respectively. These results demonstrate that the graph convolutional network, hypergraph convolutional network, and squeeze-and-excitation network make significant contributions to prediction performances.

### 3.4. Visualization Study

To interpret the capability of GHCN-SE in topology-based metabolite feature representations and the effectiveness of the squeeze-and-excitation network in feature enhancements, we conducted visualization studies through the t-distributed stochastic neighbor embedding (t-SNE) [32] and feature density values analysis on the iML1515 and Recon3D models, respectively.

**Figure 7:**
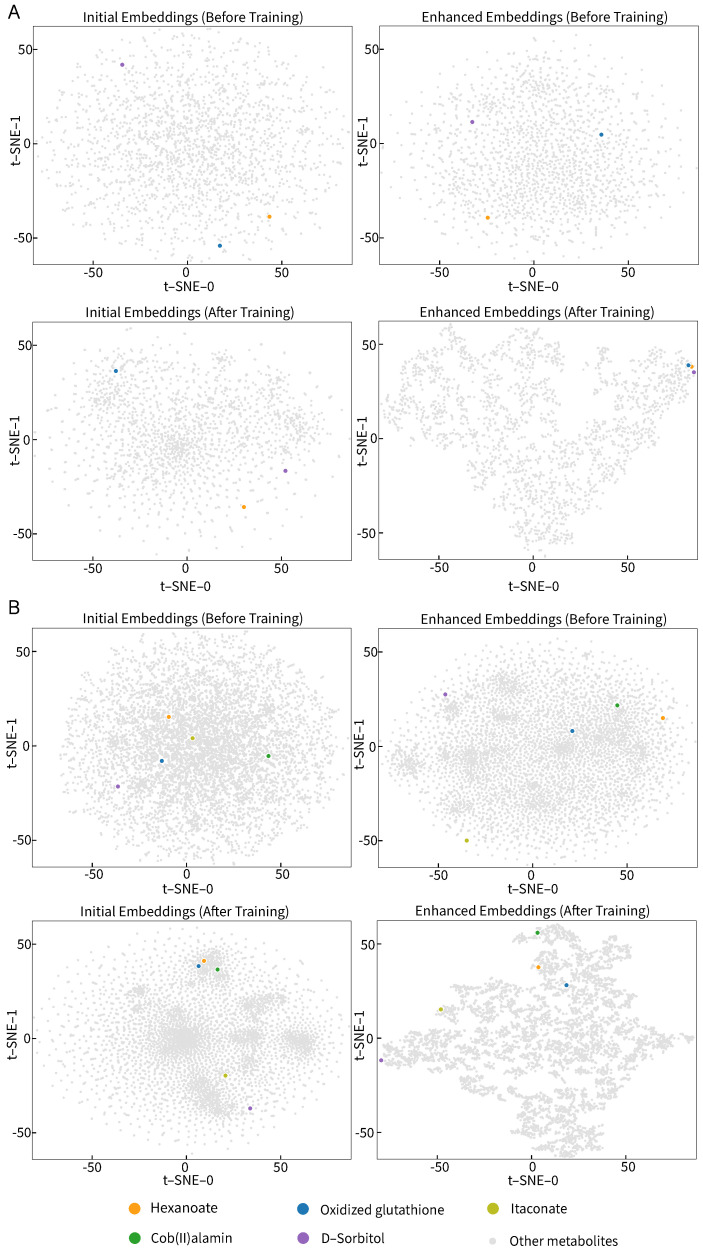
The visualization of the distributions of the initial embeddings and the enhanced embeddings before and after training using t-SNE, where the orange point denotes hexanoate, the blue point denotes oxidized glutathione, the yellow point denotes itaconate, the green point denotes cob(II)alamin, and the purple point denotes D-sorbitol. (**A**) iML1515, and (**B**) Recon3D.

We obtained the initial embeddings $x_{0,i}$ and the enhanced metabolite embeddings $z_{4,i}$ before and after training by GHCN-SE, respectively. We used t-SNE for dimensionality reduction and visualization studies on these metabolite feature embeddings. Figure 7 presents the visualization results of the distributions of the initial embeddings and the enhanced embeddings before and after training, where different colors are utilized to represent different reactions. The visualization results show that the distributions of the initial embeddings before training are completely random, and the initial features after training exhibit a mild clustering but still cannot capture the topological features of the metabolic network. The distributions of the enhanced embeddings before training are nearly random, since the model parameters have not undergone effective training at this stage. After the model training and the feature extraction and enhancement process, the distributions of the feature embeddings after feature extractions and enhancements have distinctly structured and clustered distributions. These results indicate the effectiveness of the GHCN-SE in topology-based metabolite feature representations.

**Figure 8:**
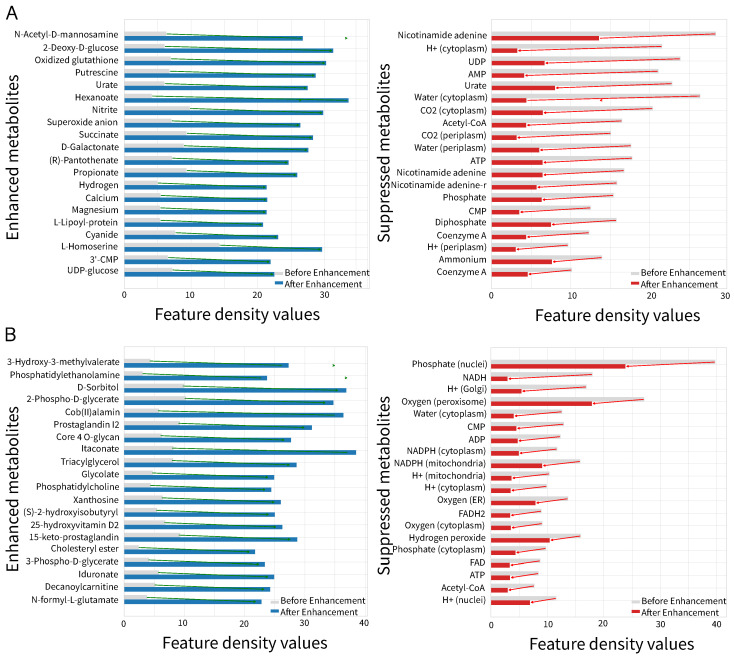
Visualization of features enhancement using the squeeze-and-excitation network for (**A**) iML1515 and (**B**) Recon3D.

Moreover, we further analyzed the metabolite feature density values before and after the squeeze-and-excitation network during the training phase. The feature density value is calculated as the $L_{2}$ norm of the metabolite feature vectors divided by the square root of the feature dimensions. Therefore, the metabolite feature densities to be compared are $\left|\right|z_{1,i}\left|\right|z_{2,i}{\left|\right|}_{2}/\sqrt{d}$ and $\left|\right|z_{4,i}{\left|\right|}_{2}/\sqrt{d}$, respectively. The feature density value can reflect the importance of metabolites in the current metabolic network. The gray bars denote the metabolite feature density values before the squeeze-and-excitation network, and the blue (left) and red (right) bars denote the feature density values after the squeeze-and-excitation network. Figure 8 shows that the squeeze-and-excitation network enhances the feature density values of some metabolites (the blue bars) and suppresses the feature density values of other some metabolites (the red bars).

The visualization results demonstrate that the squeeze-and-excitation network can achieve the feature enhancements through adaptively enhancing or suppressing the feature density values of metabolites to improve the prediction performances of GHCN-SE.

## 4. Discussion

Genome-scale metabolic models (GEMs) integrate all known genes, metabolites, and metabolic reactions into mathematical frameworks, which can reveal the association between genotype and phenotype. Gap-fillings are important for reconstructions of high-quality GEMs. Many optimization-based algorithms highly rely on phenotypic data and are difficult to apply in large-scale GEMs. Moreover, the prediction performance of these topology-based approaches by deep learning algorithms needs to be further improved.

In this study, we proposed a novel topology-based approach designated as GHCN-SE for the prediction of candidate reactions in GEMs. In the feature extraction and fusion module, GHCN-SE simultaneously employs the graph convolutional network and the hypergraph convolutional network to extract the topological features in GEMs, where the graph convolutional network can capture associations of metabolites in the same reaction and the hypergraph convolutional network can capture higher-order interactions of metabolites within reactions. GHCN-SE utilizes the learnable embeddings as metabolite initial embeddings, which can continuously be updated during training. In the feature enhancement module, we use the squeeze-and-excitation network to enhance fused metabolite features. In the output module, we aggregate the features of nodes and output the confidence scores of candidate reactions using a multi-layer perceptron.

To evaluate the reaction prediction performances and recovery performances of GHCN-SE, we conducted 5-fold cross validations on 108 high-quality BiGG GEMs for training and testing. GHCN-SE achieves the best performance compared with state-of-the-art methods across all evaluation metrics. To be specific, GHCN-SE achieves average AUPRC, recall, F1 score, accuracy, and precision scores of 0.915, 0.808, 0.816, 0.818, and 0.827, respectively, which outperform the second-best model by 0.5%, 7%, 15.4%, 2.9%, and 0.6%. Moreover, GHCN-SE achieves the best recovery performance on reaction recovery evaluations. Specifically, the average recovery rates for Top 25, Top 50, Top 100, and Top *N* achieve 0.160, 0.158, 0.152, and 0.132, which outperform the second-best model by 22.1%, 25.3%, 35.7%, and 28.2%, respectively. The ablation study was conducted to further demonstrate the contributions of the graph convolutional network, hypergraph convolutional network and squeeze-and-excitation network in GHCN-SE. Furthermore, the visualization results can demonstrate that GHCN-SE has efficient capability for metabolite feature representations and feature enhancements.

GHCN-SE significantly outperforms state-of-the-art topology-based approaches in both reaction prediction and reaction recovery experiments. Notably, GHCN-SE also surpasses the existing related approach, which integrates biochemical features of metabolites. These results demonstrate the potential of GHCN-SE for gap-fillings of draft GEMs.

Despite the remarkable improvement achieved by the GHCN-SE we proposed in reaction prediction and recovery evaluations, the results reveal potential limitations inherent to the current model. As a supervised learning framework that relies on the network topology, the performances of GHCN-SE are inevitably subject to the scale and reconstruction accuracy of draft GEMs. In future research, the biochemical information of metabolites and reactions, such as metabolite structures and enzyme information, can be considered for incorporation, which is expected to expand its potential applications in draft GEMs and constraint-based GEMs. Future research may explore methods for the designated restoration of connections between two specific metabolites.

## 5. Conclusions

In this paper, we have proposed a novel topology-based approach, named GHCN-SE, to predict confidence scores of candidate reactions and achieve gap-fillings of GEMs. GHCN-SE simultaneously employs the graph and hypergraph convolutional network to extract topological features of GEMs. The squeeze-and-excitation network is used to enhance features after the feature fusion. We evaluated the reaction prediction performances and reaction recovery performances through 5-fold cross validations on 108 high-quality BiGG GEMs. The results demonstrate that GHCN-SE achieves the best performance metrics compared with state-of-the-art deep learning-based methods. The ablation experiment demonstrates the contributions of the graph convolutional network, hypergraph convolutional network, and squeeze-and-excitation network in GHCN-SE. In addition, the visualization study demonstrates the effectiveness of GHCN-SE. GHCN-SE can be applied to improve the phenotypic prediction accuracy of draft GEMs which are generated from automated reconstruction pipelines in applications of metabolic engineering and biomedicine.

## Data Availability

The data and code presented in this study are openly available at https://github.com/kaiwang-group/GHCN-SE (accessed on 12 March 2026).

## Supplementary Materials

The following supporting information can be downloaded at: https://www.mdpi.com/article/10.3390/metabo16040258/s1. Supplementary Figure S1: The dataset partitioning and negative sample generation strategy of GHCN-SE. Supplementary Figure S2: The overall framework of GCN-SE in ablation experiments. Supplementary Figure S3: The overall framework of HCN-SE in ablation experiments. Supplementary Figure S4: The overall framework of GHCN in ablation experiments. Supplementary Figure S5: The visualization of the distributions of the initial embeddings and the enhanced embeddings before and after training using t-SNE. (A) iCHOv1, (B) RECON1. Supplementary Figure S6: The schematic diagram of construction of the graph and hypergraph. Supplementary Table S1: Hyperparameter values of constructing and training GHCN-SE. Supplementary Table S2: Hyperparameter values of CHESHIRE, HGNNP, and Multi-HGNN. Supplementary Table S3: The detailed classification of reaction numbers and categories of organism on 108 BiGG GEMs. Supplementary Table S4: The detailed 5-fold cross-validation results of reaction prediction performances of GHCN-SE on 108 BiGG GEMs. Supplementary Table S5: The detailed results of recovery rate performances of GHCN-SE on 108 BiGG GEMs.
