# Supplementary material for: A Novel Topology-Based Candidate Reaction Prediction Approach for Gap-Fillings of Genome-Scale Metabolic Models
Source: Metabolites. 2026 Apr 12;16(4):258. doi: 10.3390/metabo16040258 (PMC13118254; doi:10.3390/metabo16040258)
Supplement: Supplementary file 1 [file metabolites-16-00258-s001.zip › metabolites-4225114-supplementary.pdf]

# 1. Supplementary Figures

**Supplementary Figure S1: The dataset partitioning and negative sample generation strategy of GHCN-SE.**

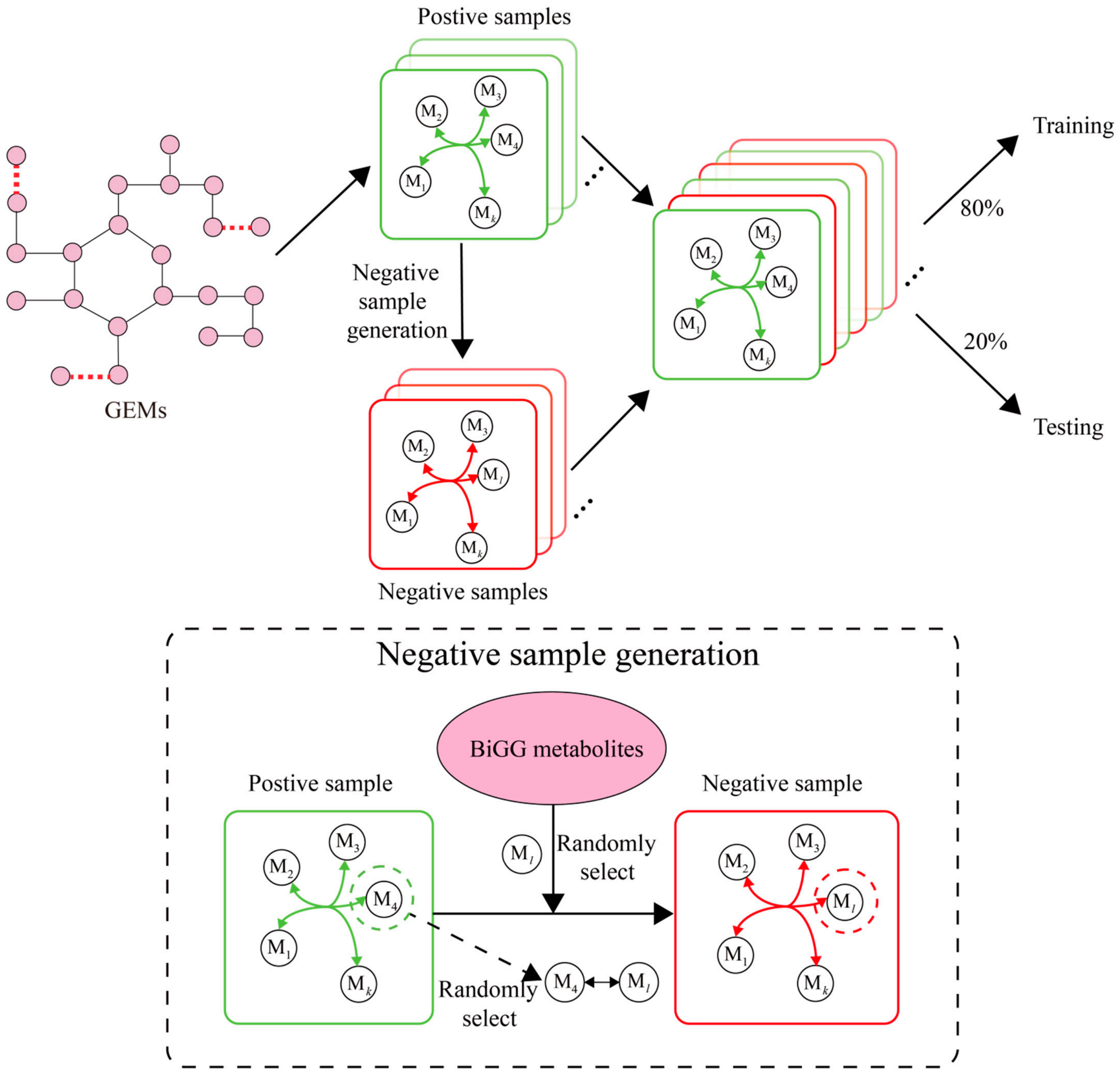

## Supplementary Figure S2: The overall framework of GCN-SE in ablation experiments.

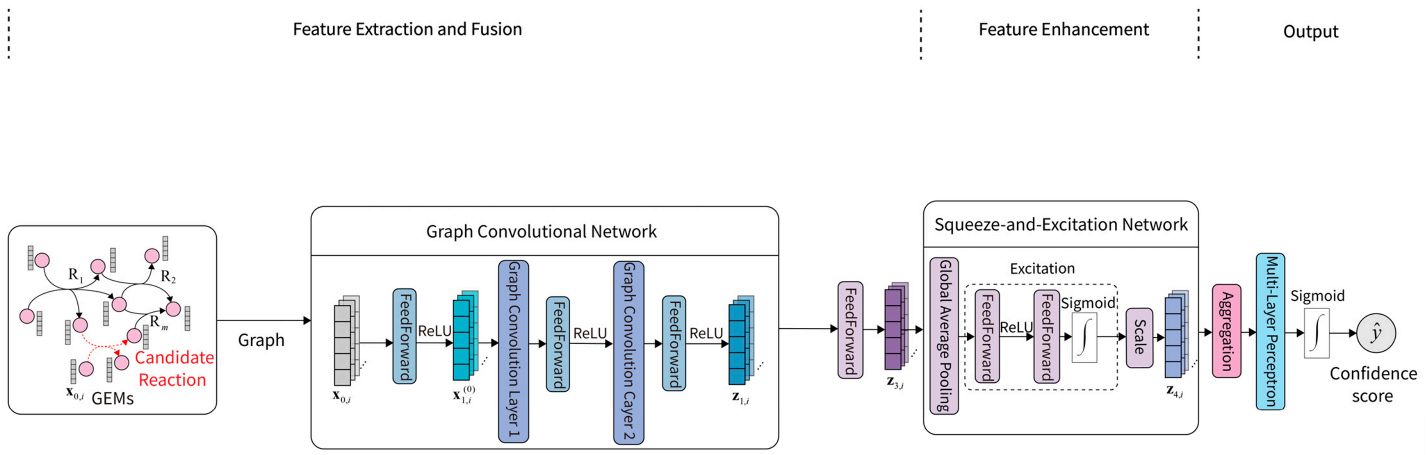

**Supplementary Figure S3: The overall framework of HCN-SE in ablation experiments.**

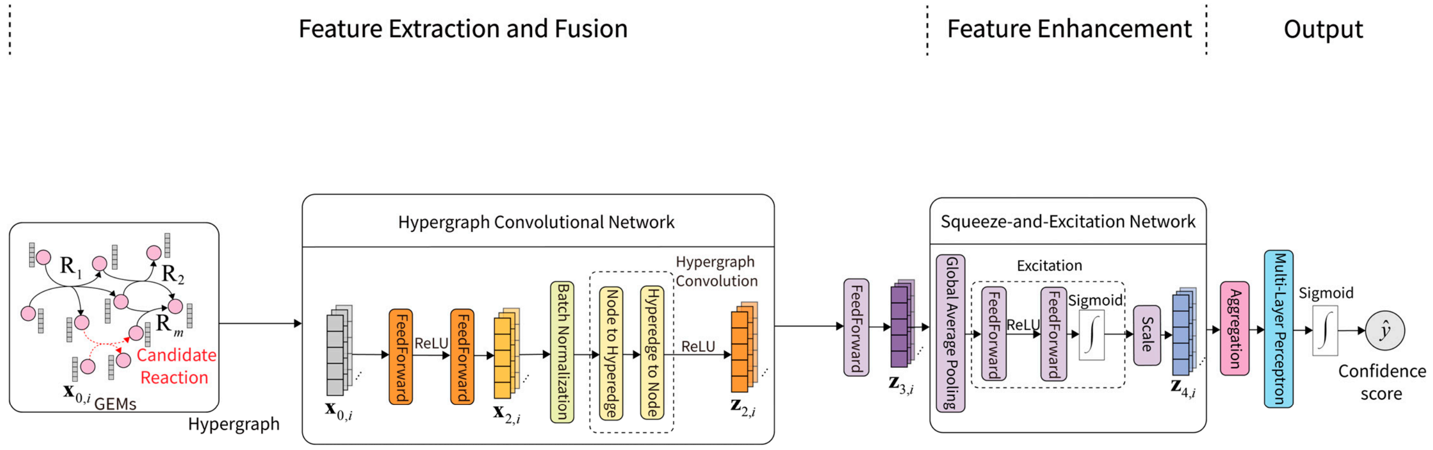

**Supplementary Figure S4: The overall framework of GHCN in ablation experiments.**

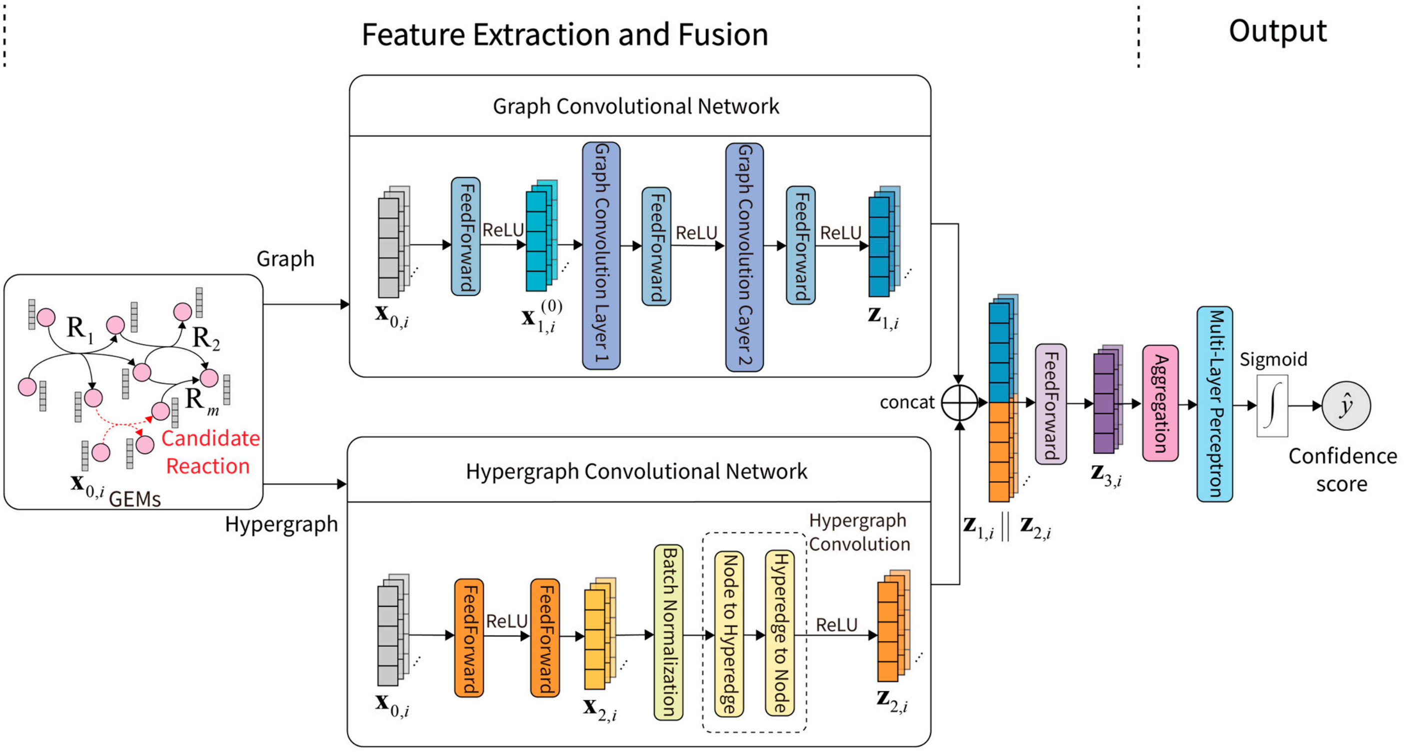

**Supplementary Figure S5: The visualization of the distributions of the initial embeddings and the enhanced embeddings before and after training using t-SNE. (A)iCHOv1, (B)RECON1.**

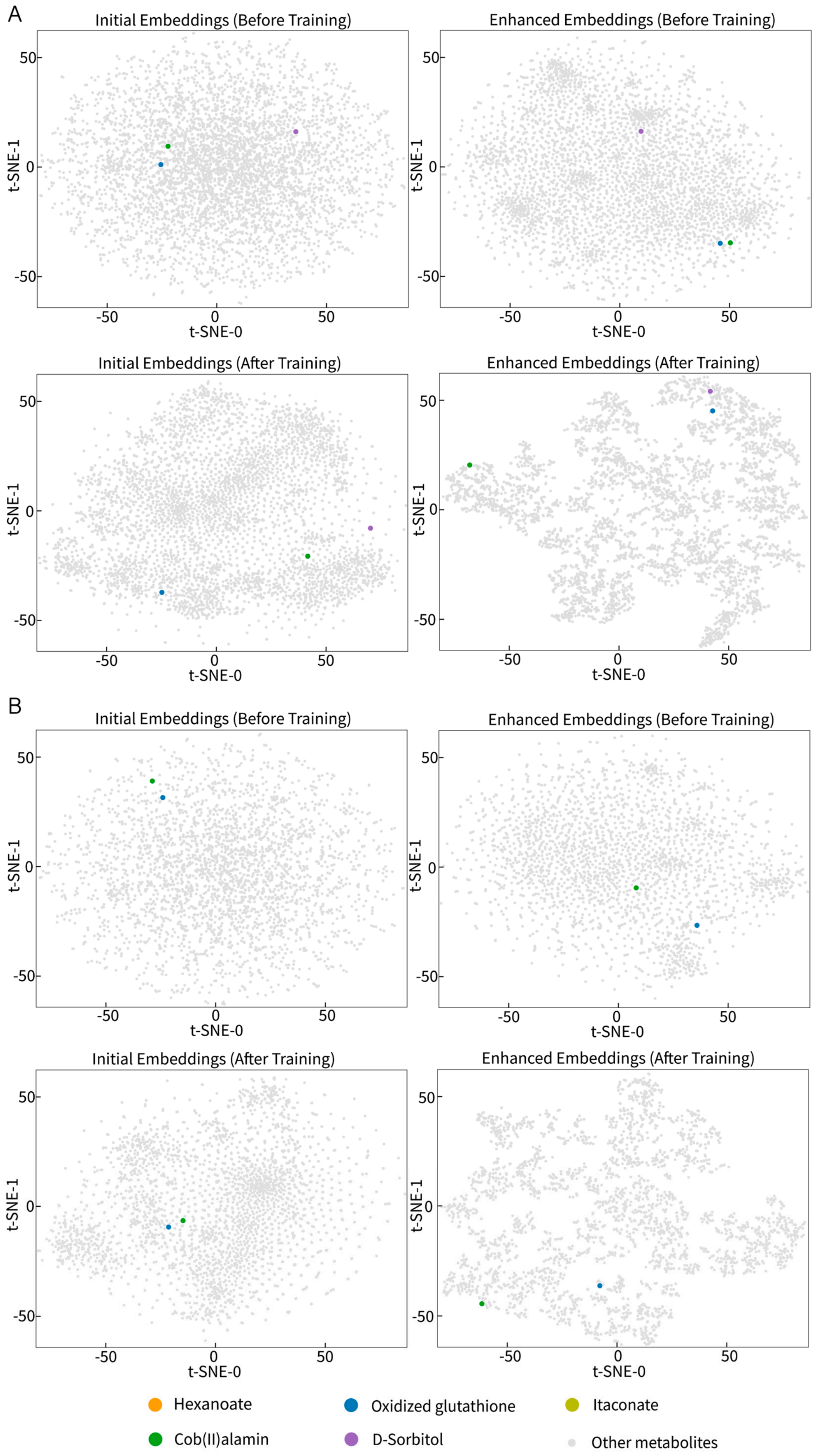

**Supplementary Figure S6: The schematic diagram of construction of the graph and hypergraph.**

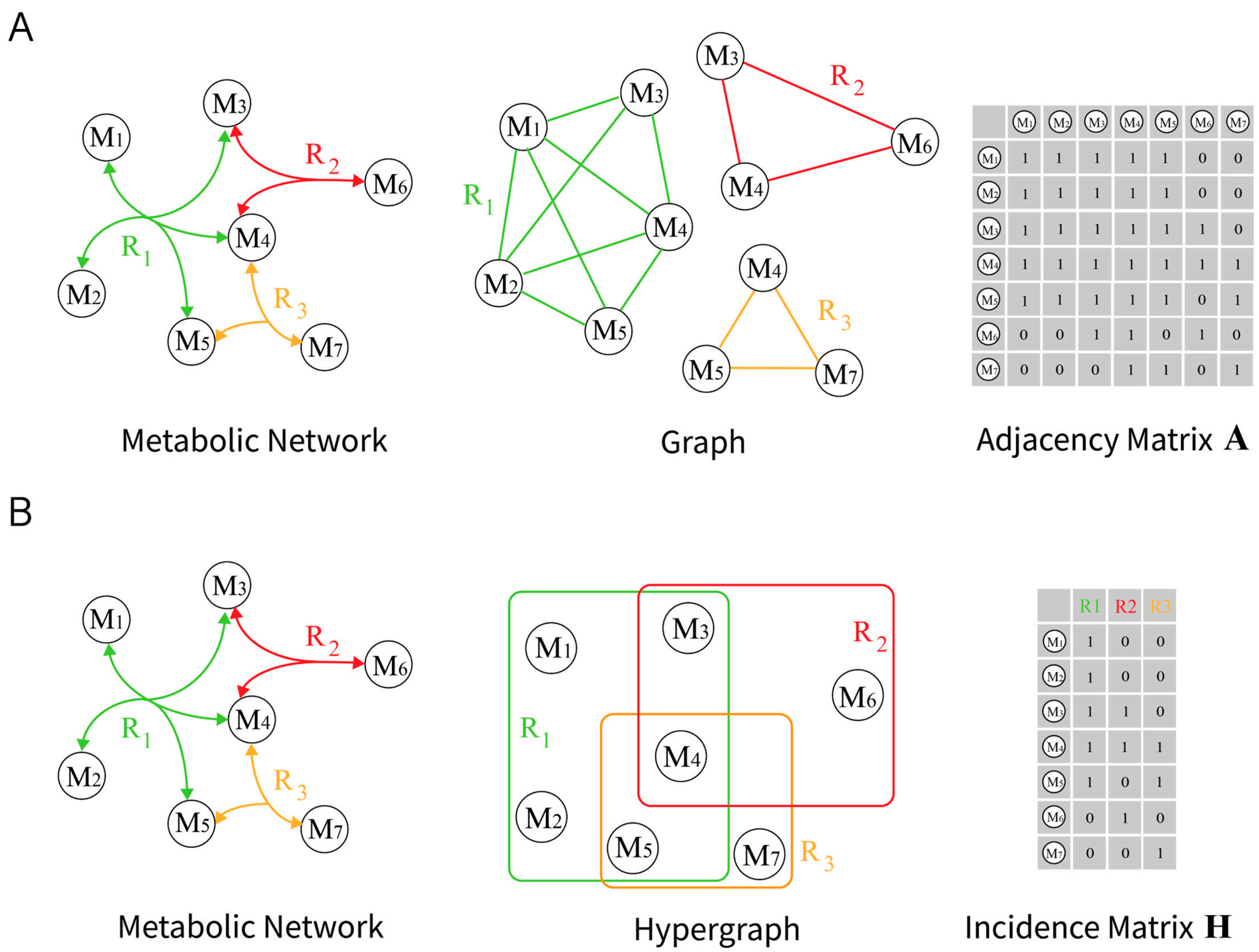

## 2.Supplementary Tables

**Supplementary Table S1: Hyperparameter values of constructing and training GHCN-SE.**

| Hyperparameters | Values |
|---|---|
| Feature dimension $N_0$ | 1024 |
| Feature dimension $N_1$ | 512 |
| Feature dimension $N_2$ | 256 |
| Feature dimension $N_3$ | 64 |
| Feature dimension $N_4$ | 16 |
| Feature dimension $N_5$ | 32 |
| Initial learning rate | 0.0005 |
| Batch sizes for different GEMs $N_{\text{batch}}$ | $16 \leq N_{\text{batch}} \leq 96$ |
| Initial feature distribution mean | 0 |
| Initial feature distribution variance | 1 |
| Dropout rate in hypergraph convolutional network | 0.5 |
| Dropout rate in output module | 0.3 |
| Epoch | 500 |

**Supplementary Table S2: Hyperparameter values of CHESHIRE, HGNNP, and Multi-HGNN.**

| Hyperparameters (CHESHIRE) | Values |
|---|---|
| Embedding dimension | 256 |
| Convolutional dimension | 128 |
| Chebyshev filter size | 3 |
| Dropout rate | 0.1 |
| Weight decay | 0.0005 |
| Initial learning rate | 0.01 |
| Batch sizes for different GEMs $N_{\text{batch}}$ | $16 \leq N_{\text{batch}} \leq 96$ |
| Epoch | 30 |

| Hyperparameters (HGNNP) | Values |
| --- | --- |
| Input dimension | 512 |
| Hidden dimension | 256 |
| Output dimension | 64 |
| Initial learning rate | 0.0005 |
| Batch sizes for different GEMs $N_{\mathrm{batch}}$ | $16 \leq N_{\mathrm{batch}} \leq 96$ |
| Dropout rate | 0.5 |
| Number of hypergraph convolutional layers | 1 |
| Epoch | 500 |

| Hyperparameters (Multi-HGNN) | Values |
|---|---|
| Input dimension | 512 |
| Hidden dimension | 256 |
| Output dimension | 64 |
| Number of directed graph layers | 2 |
| Number of hypergraph convolutional layers | 1 |
| MLP hidden layer dimension | 32 |
| Dropout rate | 0.5 |
| Weight decay | 0.0005 |
| Initial learning rate | 0.002 |
| Batch sizes for different GEMs $N_{\mathrm{batch}}$ | $16 \leq N_{\mathrm{batch}} \leq 96$ |
| Epoch | 100 |

**Supplementary Table S3: The detailed classification of reaction numbers and categories of organism on 108 BiGG GEMs.**

| GEMs | Organisms | Reactions | Categories |
|---|---|---|---|
| e_coli_core | *Escherichia coli* str. K-12 substr. MG1655 | 95 | prokaryote |
| iAB_RBC_283 | *Homo sapiens* | 469 | eukaryote |
| iIS312 | *Trypanosoma cruzi* Dm28c | 519 | eukaryote |
| iIS312_Amastigote | *Trypanosoma cruzi* Dm28c | 519 | eukaryote |
| iIS312_Epimastigote | *Trypanosoma cruzi* Dm28c | 519 | eukaryote |
| iIS312_Trypomastigote | *Trypanosoma cruzi* Dm28c | 520 | eukaryote |
| iIT341 | *Helicobacter pylori* 26695 | 554 | prokaryote |
| iLJ478 | *Thermotoga maritima* MSB8 | 652 | prokaryote |
| iAF692 | *Methanosarcina barkeri* str. Fusaro | 690 | prokaryote |
| iSB619 | *Staphylococcus aureus* subsp. aureus N315 | 743 | prokaryote |
| iNF517 | *Lactococcus lactis* subsp. cremoris MG1363 | 754 | prokaryote |
| iHN637 | *Clostridium ljungdahlii* DSM 13528 | 785 | prokaryote |
| iJB785 | *Synechococcus elongatus* PCC 7942 | 849 | prokaryote |
| iJN678 | *Synechocystis* sp. PCC 6803 | 863 | prokaryote |
| iAT_PLT_636 | *Homo sapiens* | 1008 | eukaryote |
| iCN718 | *Acinetobacter baumannii* AYE | 1015 | prokaryote |
| iNJ661 | *Mycobacterium tuberculosis* H37Rv | 1025 | prokaryote |
| iSynCJ816 | *Synechocystis* sp. PCC 6803 | 1044 | prokaryote |
| iJN746 | *Pseudomonas putida* KT2440 | 1054 | prokaryote |
| iAM_Pb448 | *Plasmodium berghei* | 1067 | eukaryote |
| iAM_Pc455 | *Plasmodium cynomolgi* strain B | 1074 | eukaryote |
| iJR904 | *Escherichia coli* str. K-12 substr. MG1655 | 1075 | prokaryote |
| iAM_Pv461 | *Plasmodium vivax* Sal-1 | 1078 | eukaryote |
| iAM_Pk459 | *Plasmodium knowlesi* strain H | 1079 | eukaryote |
| iAM_Pf480 | *Plasmodium falciparum* 3D7 | 1083 | eukaryote |
| iEK1008 | *Mycobacterium tuberculosis* H37Rv | 1226 | prokaryote |
| iCN900 | *Clostridioides difficile* 630 | 1229 | prokaryote |
| iYO844 | *Bacillus subtilis* subsp. subtilis str. 168 | 1250 | prokaryote |

| GEMs | Organisms | Reactions | Categories |
|---|---|---|---|
| iND750 | *Saccharomyces cerevisiae* S288C | 1266 | eukaryotes |
| iAF987 | *Geobacter metallireducens* GS-15 | 1285 | prokaryotes |
| iYS854 | *Staphylococcus aureus* subsp. aureus USA300_TCH1516 | 1455 | prokaryotes |
| iMM904 | *Saccharomyces cerevisiae* S288C | 1577 | eukaryotes |
| iPC815 | *Yersinia pestis* CO92 | 1961 | prokaryotes |
| iRC1080 | *Chlamydomonas reinhardtii* | 2191 | eukaryotes |
| iYL1228 | *Klebsiella pneumoniae* subsp. pneumoniae MGH 78578 | 2262 | prokaryotes |
| iAF1260 | *Escherichia coli* str. K-12 substr. MG1655 | 2382 | prokaryotes |
| iAF1260b | *Escherichia coli* str. K-12 substr. MG1655 | 2388 | prokaryotes |
| iSDY_1059 | *Shigella dysenteriae* Sd197 | 2539 | prokaryotes |
| STM_v1_0 | *Salmonella enterica* subsp. enterica serovar Typhimurium str. LT2 | 2545 | prokaryotes |
| iJO1366 | *Escherichia coli* str. K-12 substr. MG1655 | 2583 | prokaryotes |
| iSbBS512_1146 | *Shigella boydii* CDC 3083-94 | 2591 | prokaryotes |
| iSBO_1134 | *Shigella boydii* Sb227 | 2591 | prokaryotes |
| iS_1188 | *Shigella flexneri* 2a str. 2457T | 2619 | prokaryotes |
| iSFV_1184 | *Shigella flexneri* 5 str. 8401 | 2621 | prokaryotes |
| iSF_1195 | *Shigella flexneri* 2a str. 301 | 2630 | prokaryotes |
| iSFxv_1172 | *Shigella flexneri* 2002017 | 2638 | prokaryotes |
| iSSON_1240 | *Shigella sonnei* Ss046 | 2693 | prokaryotes |
| iECH74115_1262 | *Escherichia coli* O157:H7 str. EC4115 | 2694 | prokaryotes |
| iE2348C_1286 | *Escherichia coli* O127:H6 str. E2348/69 | 2703 | prokaryotes |
| iG2583_1286 | *Escherichia coli* O55:H7 str. CB9615 | 2704 | prokaryotes |
| iECED1_1282 | *Escherichia coli* ED1a | 2706 | prokaryotes |
| iECSP_1301 | *Escherichia coli* O157:H7 str. TW14359 | 2712 | prokaryotes |
| iML1515 | *Escherichia coli* str. K-12 substr. MG1655 | 2712 | prokaryotes |
| iEC042_1314 | *Escherichia coli* 042 | 2714 | prokaryotes |
| iECNA114_1301 | *Escherichia coli* NA114 | 2718 | prokaryotes |
| iECs_1301 | *Escherichia coli* O157:H7 str. Sakai | 2720 | prokaryotes |
| iECIAI39_1322 | *Escherichia coli* IAI39 | 2721 | prokaryotes |

| GEMs | Organisms | Reactions | Categories |
|---|---|---|---|
| iZ_1308 | *Escherichia coli* O157:H7 str. EDL933 | 2721 | prokaryotes |
| iUTI89_1310 | *Escherichia coli* UTI89 | 2725 | prokaryotes |
| ic_1306 | *Escherichia coli* CFT073 | 2726 | prokaryotes |
| iEC1344_C | *Escherichia coli* C | 2726 | prokaryotes |
| iLF82_1304 | *Escherichia coli* LF82 | 2726 | prokaryotes |
| iECOK1_1307 | *Escherichia coli* IHE3034 | 2729 | prokaryotes |
| iECS88_1305 | *Escherichia coli* S88 | 2729 | prokaryotes |
| iECABU_c1320 | *Escherichia coli* ABU 83972 | 2731 | prokaryotes |
| iAPECO1_1312 | *Escherichia coli* APEC O1 | 2735 | prokaryotes |
| iNRG857_1313 | *Escherichia coli* O83:H1 str. NRG 857C | 2735 | prokaryotes |
| iUMN146_1321 | *Escherichia coli* UM146 | 2735 | prokaryotes |
| iECP_1309 | *Escherichia coli* 536 | 2739 | prokaryotes |
| iEC1356_Bl21DE3 | *Escherichia coli* BL21(DE3) | 2740 | prokaryotes |
| iECUMN_1333 | *Escherichia coli* UMN026 | 2740 | prokaryotes |
| iB21_1397 | *Escherichia coli* BL21(DE3) | 2741 | prokaryotes |
| iBWG_1329 | *Escherichia coli* BW2952 | 2741 | prokaryotes |
| iECD_1391 | *Escherichia coli* BL21(DE3) | 2741 | prokaryotes |
| iECDH10B_1368 | *Escherichia coli* str. K-12 substr. DH10B | 2742 | prokaryotes |
| iECSF_1327 | *Escherichia coli* SE15 | 2742 | prokaryotes |
| iEcSMS35_1347 | *Escherichia coli* SMS-3-5 | 2746 | prokaryotes |
| iECB_1328 | *Escherichia coli* B str. REL606 | 2748 | prokaryotes |
| iECBD_1354 | *Escherichia coli* 'BL21-Gold(DE3)pLysS AG' | 2748 | prokaryotes |
| iEcDH1_1363 | *Escherichia coli* DH1 | 2750 | prokaryotes |
| iEcHS_1320 | *Escherichia coli* HS | 2753 | prokaryotes |
| iECDH1ME8569_1439 | *Escherichia coli* DH1 | 2755 | prokaryotes |
| iEC1349_Crooks | *Escherichia coli* ATCC 8739 | 2756 | prokaryotes |
| iEC55989_1330 | *Escherichia coli* 55989 | 2756 | prokaryotes |
| iETEC_1333 | *Escherichia coli* ETEC H10407 | 2756 | prokaryotes |
| iEC1372_W3110 | *Escherichia coli* str. K-12 substr. W3110 | 2758 | prokaryotes |
| iECO103_1326 | *Escherichia coli* O103:H2 str. 12009 | 2758 | prokaryotes |

| GEMs | Organisms | Reactions | Categories |
|---|---|---|---|
| iY75_1357 | *Escherichia coli* str. K-12 substr. W3110 | 2759 | prokaryotes |
| iECO111_1330 | *Escherichia coli* O111:H- str. 11128 | 2760 | prokaryotes |
| iEcE24377_1341 | *Escherichia coli* O139:H28 str. E24377A | 2763 | prokaryotes |
| iEC1364_W | *Escherichia coli* W | 2764 | prokaryotes |
| iECIAI1_1343 | *Escherichia coli* IAI1 | 2765 | prokaryotes |
| iEcolC_1368 | *Escherichia coli* ATCC 8739 | 2768 | prokaryotes |
| iECSE_1348 | *Escherichia coli* SE11 | 2768 | prokaryotes |
| iUMNK88_1353 | *Escherichia coli* UMNK88 | 2777 | prokaryotes |
| iEKO11_1354 | *Escherichia coli* KO11FL | 2778 | prokaryotes |
| iEC1368_DH5a | *Escherichia coli* DH5[alpha] | 2779 | prokaryotes |
| iECO26_1355 | *Escherichia coli* O26:H11 str. 11368 | 2780 | prokaryotes |
| iECW_1372 | *Escherichia coli* W | 2782 | prokaryotes |
| iWFL_1372 | *Escherichia coli* W | 2782 | prokaryotes |
| iJN1463 | *Pseudomonas putida* KT2440 | 2927 | prokaryotes |
| iYS1720 | *Salmonella pan-reactome* | 3357 | prokaryotes |
| iMM1415 | *Mus musculus* | 3726 | eukaryotes |
| RECON1 | *Homo sapiens* | 3741 | eukaryotes |
| iCHOv1_DG44 | *Cricetulus griseus* | 3942 | eukaryotes |
| iLB1027_lipid | *Phaeodactylum tricornutum* CCAP 1055/1 | 4456 | eukaryotes |
| iCHOv1 | *Cricetulus griseus* | 6663 | eukaryotes |
| Recon3D | *Homo sapiens* | 10600 | eukaryotes |

**Supplementary Table S4: The detailed 5-fold cross-validation results of reaction prediction performances of GHCN-SE on 108 BiGG GEMs.**

| GEMs | AUPRC (fold 0) | AUPRC (fold 1) | AUPRC (fold 2) | AUPRC (fold 3) | AUPRC (fold 4) | AUPRC (average) |
|---|---|---|---|---|---|---|
| RECON1 | 0.926 | 0.918 | 0.908 | 0.926 | 0.907 | 0.917 |
| Recon3D | 0.941 | 0.936 | 0.924 | 0.935 | 0.930 | 0.933 |
| STM_v1_0 | 0.919 | 0.918 | 0.910 | 0.901 | 0.906 | 0.911 |
| e_coli_core | 0.743 | 0.860 | 0.746 | 0.762 | 0.863 | 0.795 |
| iAB_RBC_283 | 0.926 | 0.859 | 0.870 | 0.877 | 0.901 | 0.887 |
| iAF1260 | 0.912 | 0.915 | 0.936 | 0.929 | 0.905 | 0.919 |
| iAF1260b | 0.902 | 0.916 | 0.926 | 0.923 | 0.921 | 0.918 |
| iAF692 | 0.942 | 0.941 | 0.942 | 0.940 | 0.928 | 0.939 |
| iAF987 | 0.963 | 0.935 | 0.942 | 0.961 | 0.949 | 0.950 |
| iAM_Pb448 | 0.876 | 0.903 | 0.883 | 0.850 | 0.908 | 0.884 |
| iAM_Pc455 | 0.894 | 0.908 | 0.896 | 0.870 | 0.873 | 0.888 |
| iAM_Pf480 | 0.890 | 0.888 | 0.872 | 0.888 | 0.878 | 0.883 |
| iAM_Pk459 | 0.855 | 0.890 | 0.880 | 0.862 | 0.887 | 0.875 |
| iAM_Pv461 | 0.888 | 0.846 | 0.860 | 0.876 | 0.901 | 0.874 |
| iAPECO1_1312 | 0.905 | 0.909 | 0.920 | 0.914 | 0.917 | 0.913 |
| iAT_PLT_636 | 0.876 | 0.914 | 0.932 | 0.933 | 0.932 | 0.917 |
| iB21_1397 | 0.924 | 0.911 | 0.914 | 0.922 | 0.936 | 0.921 |
| iBWG_1329 | 0.921 | 0.918 | 0.912 | 0.914 | 0.917 | 0.916 |
| iCHOv1 | 0.961 | 0.954 | 0.958 | 0.953 | 0.963 | 0.958 |
| iCHOv1_DG44 | 0.903 | 0.888 | 0.895 | 0.895 | 0.895 | 0.895 |
| iCN718 | 0.928 | 0.947 | 0.928 | 0.925 | 0.947 | 0.935 |
| iCN900 | 0.925 | 0.955 | 0.922 | 0.950 | 0.956 | 0.942 |
| iE2348C_1286 | 0.916 | 0.917 | 0.909 | 0.920 | 0.924 | 0.917 |
| iEC042_1314 | 0.923 | 0.910 | 0.891 | 0.889 | 0.902 | 0.903 |
| iEC1344_C | 0.926 | 0.912 | 0.928 | 0.913 | 0.916 | 0.919 |
| iEC1349_Crooks | 0.929 | 0.910 | 0.927 | 0.901 | 0.942 | 0.922 |
| iEC1356_Bl21DE3 | 0.907 | 0.927 | 0.918 | 0.913 | 0.916 | 0.916 |
| iEC1364_W | 0.939 | 0.912 | 0.919 | 0.926 | 0.934 | 0.926 |
| iEC1368_DH5a | 0.916 | 0.925 | 0.932 | 0.923 | 0.924 | 0.924 |
| iEC1372_W3110 | 0.937 | 0.923 | 0.918 | 0.921 | 0.926 | 0.925 |
| iEC55989_1330 | 0.930 | 0.920 | 0.906 | 0.911 | 0.913 | 0.916 |
| iECABU_c1320 | 0.902 | 0.906 | 0.913 | 0.901 | 0.901 | 0.904 |
| iECBD_1354 | 0.913 | 0.916 | 0.911 | 0.918 | 0.930 | 0.918 |
| iECB_1328 | 0.919 | 0.922 | 0.916 | 0.918 | 0.925 | 0.920 |
| iECDH10B_1368 | 0.920 | 0.900 | 0.916 | 0.895 | 0.924 | 0.911 |
| iECDH1ME8569_1439 | 0.915 | 0.926 | 0.903 | 0.921 | 0.915 | 0.916 |
| iECD_1391 | 0.900 | 0.918 | 0.926 | 0.922 | 0.922 | 0.917 |
| iECED1_1282 | 0.919 | 0.912 | 0.923 | 0.903 | 0.920 | 0.915 |
| iECH74115_1262 | 0.920 | 0.913 | 0.909 | 0.903 | 0.917 | 0.912 |
| iECIAI1_1343 | 0.927 | 0.915 | 0.910 | 0.897 | 0.918 | 0.913 |
| iECIAI39_1322 | 0.911 | 0.905 | 0.912 | 0.906 | 0.916 | 0.910 |

| GEMs | AUPRC (fold 0) | AUPRC (fold 1) | AUPRC (fold 2) | AUPRC (fold 3) | AUPRC (fold 4) | AUPRC (average) |
|---|---|---|---|---|---|---|
| iECNA114_1301 | 0.917 | 0.922 | 0.920 | 0.910 | 0.914 | 0.917 |
| iECO103_1326 | 0.927 | 0.920 | 0.916 | 0.933 | 0.927 | 0.925 |
| iECO111_1330 | 0.920 | 0.908 | 0.905 | 0.913 | 0.922 | 0.913 |
| iECO26_1355 | 0.920 | 0.908 | 0.907 | 0.911 | 0.928 | 0.915 |
| iECOK1_1307 | 0.904 | 0.907 | 0.927 | 0.911 | 0.916 | 0.913 |
| iECP_1309 | 0.929 | 0.907 | 0.910 | 0.917 | 0.917 | 0.916 |
| iECS88_1305 | 0.914 | 0.910 | 0.903 | 0.908 | 0.899 | 0.907 |
| iECSE_1348 | 0.913 | 0.930 | 0.913 | 0.903 | 0.936 | 0.919 |
| iECSF_1327 | 0.895 | 0.905 | 0.916 | 0.906 | 0.902 | 0.905 |
| iECSP_1301 | 0.918 | 0.904 | 0.914 | 0.931 | 0.921 | 0.917 |
| iECUMN_1333 | 0.921 | 0.906 | 0.907 | 0.914 | 0.930 | 0.916 |
| iECW_1372 | 0.917 | 0.940 | 0.917 | 0.923 | 0.935 | 0.926 |
| iECs_1301 | 0.885 | 0.909 | 0.911 | 0.907 | 0.931 | 0.908 |
| iEK1008 | 0.963 | 0.961 | 0.968 | 0.956 | 0.937 | 0.957 |
| iEKO11_1354 | 0.910 | 0.932 | 0.910 | 0.921 | 0.926 | 0.920 |
| iETEC_1333 | 0.911 | 0.907 | 0.927 | 0.919 | 0.919 | 0.916 |
| iEcDH1_1363 | 0.909 | 0.932 | 0.924 | 0.919 | 0.938 | 0.925 |
| iEcE24377_1341 | 0.904 | 0.916 | 0.909 | 0.937 | 0.926 | 0.918 |
| iEcHS_1320 | 0.918 | 0.910 | 0.900 | 0.928 | 0.929 | 0.917 |
| iEcSMS35_1347 | 0.915 | 0.924 | 0.919 | 0.915 | 0.919 | 0.918 |
| iEcolC_1368 | 0.904 | 0.910 | 0.921 | 0.928 | 0.914 | 0.915 |
| iG2583_1286 | 0.919 | 0.915 | 0.893 | 0.914 | 0.919 | 0.912 |
| iHN637 | 0.932 | 0.915 | 0.923 | 0.937 | 0.945 | 0.930 |
| iIS312 | 0.847 | 0.885 | 0.830 | 0.861 | 0.867 | 0.858 |
| iIS312_Amastigote | 0.868 | 0.807 | 0.822 | 0.880 | 0.887 | 0.853 |
| iIS312_Epimastigote | 0.877 | 0.833 | 0.869 | 0.770 | 0.857 | 0.841 |
| iIS312_Trypomastigote | 0.867 | 0.850 | 0.882 | 0.861 | 0.845 | 0.861 |
| iIT341 | 0.876 | 0.925 | 0.904 | 0.899 | 0.883 | 0.897 |
| iJB785 | 0.944 | 0.959 | 0.971 | 0.942 | 0.924 | 0.948 |
| iJN1463 | 0.909 | 0.934 | 0.937 | 0.930 | 0.937 | 0.930 |
| iJN678 | 0.960 | 0.971 | 0.964 | 0.967 | 0.919 | 0.956 |
| iJN746 | 0.929 | 0.934 | 0.953 | 0.944 | 0.933 | 0.939 |
| iJO1366 | 0.930 | 0.905 | 0.909 | 0.917 | 0.898 | 0.911 |
| iJR904 | 0.930 | 0.930 | 0.927 | 0.926 | 0.927 | 0.928 |
| iLB1027_lipid | 0.988 | 0.987 | 0.987 | 0.985 | 0.988 | 0.987 |
| iLF82_1304 | 0.917 | 0.908 | 0.882 | 0.928 | 0.923 | 0.911 |
| iLJ478 | 0.938 | 0.960 | 0.932 | 0.931 | 0.927 | 0.938 |
| iML1515 | 0.919 | 0.914 | 0.930 | 0.922 | 0.924 | 0.922 |
| iMM1415 | 0.909 | 0.922 | 0.895 | 0.914 | 0.910 | 0.910 |
| iMM904 | 0.911 | 0.870 | 0.880 | 0.912 | 0.906 | 0.895 |
| iND750 | 0.905 | 0.896 | 0.895 | 0.920 | 0.939 | 0.911 |
| iNF517 | 0.910 | 0.939 | 0.936 | 0.945 | 0.926 | 0.931 |

| GEMs | AUPRC (fold 0) | AUPRC (fold 1) | AUPRC (fold 2) | AUPRC (fold 3) | AUPRC (fold 4) | AUPRC (average) |
|---|---|---|---|---|---|---|
| iNJ661 | 0.952 | 0.964 | 0.961 | 0.942 | 0.952 | 0.954 |
| iNRG857_1313 | 0.916 | 0.915 | 0.905 | 0.881 | 0.930 | 0.909 |
| iPC815 | 0.904 | 0.909 | 0.876 | 0.908 | 0.907 | 0.901 |
| iRC1080 | 0.956 | 0.949 | 0.957 | 0.930 | 0.930 | 0.944 |
| iSB619 | 0.941 | 0.924 | 0.922 | 0.939 | 0.949 | 0.935 |
| iSBO_1134 | 0.907 | 0.927 | 0.896 | 0.899 | 0.932 | 0.912 |
| iSDY_1059 | 0.926 | 0.889 | 0.918 | 0.901 | 0.941 | 0.915 |
| iSFV_1184 | 0.904 | 0.900 | 0.898 | 0.904 | 0.898 | 0.901 |
| iSF_1195 | 0.920 | 0.907 | 0.926 | 0.920 | 0.922 | 0.919 |
| iSFxv_1172 | 0.906 | 0.891 | 0.890 | 0.904 | 0.923 | 0.903 |
| iSSON_1240 | 0.932 | 0.913 | 0.898 | 0.910 | 0.908 | 0.912 |
| iS_1188 | 0.920 | 0.894 | 0.919 | 0.906 | 0.902 | 0.908 |
| iSbBS512_1146 | 0.915 | 0.906 | 0.906 | 0.883 | 0.893 | 0.900 |
| iSynCJ816 | 0.943 | 0.913 | 0.960 | 0.957 | 0.948 | 0.944 |
| iUMN146_1321 | 0.917 | 0.894 | 0.919 | 0.917 | 0.914 | 0.912 |
| iUMNK88_1353 | 0.913 | 0.920 | 0.910 | 0.912 | 0.915 | 0.914 |
| iUTI89_1310 | 0.917 | 0.923 | 0.916 | 0.904 | 0.931 | 0.918 |
| iWFL_1372 | 0.919 | 0.933 | 0.890 | 0.920 | 0.924 | 0.917 |
| iY75_1357 | 0.920 | 0.910 | 0.920 | 0.896 | 0.922 | 0.914 |
| iYL1228 | 0.945 | 0.937 | 0.916 | 0.952 | 0.907 | 0.931 |
| iYO844 | 0.943 | 0.937 | 0.925 | 0.949 | 0.930 | 0.937 |
| iYS1720 | 0.927 | 0.909 | 0.915 | 0.892 | 0.909 | 0.910 |
| iYS854 | 0.892 | 0.898 | 0.902 | 0.904 | 0.922 | 0.904 |
| iZ_1308 | 0.913 | 0.923 | 0.917 | 0.912 | 0.916 | 0.916 |
| ic_1306 | 0.896 | 0.915 | 0.907 | 0.924 | 0.929 | 0.914 |

| GEMs | Recall (fold 0) | Recall (fold 1) | Recall (fold 2) | Recall (fold 3) | Recall (fold 4) | Recall (average) |
|---|---|---|---|---|---|---|
| RECON1 | 0.830 | 0.838 | 0.818 | 0.830 | 0.821 | 0.828 |
| Recon3D | 0.816 | 0.835 | 0.785 | 0.808 | 0.805 | 0.810 |
| STM_v1_0 | 0.802 | 0.779 | 0.781 | 0.850 | 0.800 | 0.803 |
| e_coli_core | 0.438 | 0.875 | 0.750 | 0.625 | 0.875 | 0.713 |
| iAB_RBC_283 | 0.891 | 0.766 | 0.797 | 0.891 | 0.844 | 0.838 |
| iAF1260 | 0.810 | 0.813 | 0.852 | 0.867 | 0.836 | 0.835 |
| iAF1260b | 0.818 | 0.818 | 0.841 | 0.849 | 0.810 | 0.827 |
| iAF692 | 0.875 | 0.917 | 0.802 | 0.708 | 0.688 | 0.798 |
| iAF987 | 0.870 | 0.896 | 0.870 | 0.870 | 0.901 | 0.881 |
| iAM_Pb448 | 0.734 | 0.771 | 0.839 | 0.708 | 0.755 | 0.761 |
| iAM_Pc455 | 0.672 | 0.766 | 0.615 | 0.792 | 0.719 | 0.713 |
| iAM_Pf480 | 0.776 | 0.849 | 0.760 | 0.854 | 0.792 | 0.806 |
| iAM_Pk459 | 0.818 | 0.740 | 0.813 | 0.719 | 0.776 | 0.773 |
| iAM_Pv461 | 0.792 | 0.729 | 0.776 | 0.755 | 0.781 | 0.767 |
| iAPECO1_1312 | 0.790 | 0.798 | 0.663 | 0.846 | 0.794 | 0.778 |
| iAT_PLT_636 | 0.776 | 0.813 | 0.833 | 0.854 | 0.807 | 0.817 |
| iB21_1397 | 0.790 | 0.790 | 0.802 | 0.794 | 0.783 | 0.792 |
| iBWG_1329 | 0.817 | 0.800 | 0.760 | 0.773 | 0.835 | 0.797 |
| iCHOv1 | 0.874 | 0.838 | 0.859 | 0.841 | 0.887 | 0.860 |
| iCHOv1_DG44 | 0.783 | 0.764 | 0.770 | 0.780 | 0.775 | 0.774 |
| iCN718 | 0.740 | 0.792 | 0.771 | 0.693 | 0.766 | 0.752 |
| iCN900 | 0.771 | 0.885 | 0.823 | 0.859 | 0.906 | 0.849 |
| iE2348C_1286 | 0.806 | 0.831 | 0.823 | 0.813 | 0.825 | 0.820 |
| iEC042_1314 | 0.794 | 0.627 | 0.821 | 0.840 | 0.858 | 0.788 |
| iEC1344_C | 0.833 | 0.806 | 0.848 | 0.835 | 0.835 | 0.832 |
| iEC1349_Crooks | 0.808 | 0.819 | 0.821 | 0.775 | 0.827 | 0.810 |
| iEC1356_Bl21DE3 | 0.838 | 0.792 | 0.796 | 0.852 | 0.846 | 0.825 |
| iEC1364_W | 0.863 | 0.825 | 0.827 | 0.846 | 0.848 | 0.842 |
| iEC1368_DH5a | 0.802 | 0.835 | 0.827 | 0.850 | 0.833 | 0.830 |
| iEC1372_W3110 | 0.842 | 0.769 | 0.848 | 0.838 | 0.773 | 0.814 |
| iEC55989_1330 | 0.848 | 0.775 | 0.790 | 0.823 | 0.800 | 0.807 |
| iECABU_c1320 | 0.796 | 0.825 | 0.806 | 0.800 | 0.813 | 0.808 |
| iECBD_1354 | 0.810 | 0.788 | 0.865 | 0.827 | 0.848 | 0.828 |
| iECB_1328 | 0.806 | 0.802 | 0.821 | 0.806 | 0.835 | 0.814 |
| iECDH10B_1368 | 0.752 | 0.785 | 0.819 | 0.817 | 0.844 | 0.803 |
| iECDH1ME8569_1439 | 0.656 | 0.746 | 0.779 | 0.827 | 0.813 | 0.764 |
| iECD_1391 | 0.817 | 0.810 | 0.817 | 0.842 | 0.829 | 0.823 |
| iECED1_1282 | 0.779 | 0.835 | 0.783 | 0.727 | 0.779 | 0.781 |
| iECH74115_1262 | 0.769 | 0.825 | 0.835 | 0.688 | 0.815 | 0.786 |
| iECIAI1_1343 | 0.800 | 0.823 | 0.804 | 0.796 | 0.821 | 0.809 |
| iECIAI39_1322 | 0.796 | 0.740 | 0.810 | 0.804 | 0.788 | 0.788 |
| iECNA114_1301 | 0.810 | 0.825 | 0.758 | 0.823 | 0.829 | 0.809 |
| iECO103_1326 | 0.856 | 0.827 | 0.829 | 0.829 | 0.792 | 0.827 |
| iECO111_1330 | 0.796 | 0.804 | 0.756 | 0.829 | 0.831 | 0.803 |

| GEMs | Recall (fold 0) | Recall (fold 1) | Recall (fold 2) | Recall (fold 3) | Recall (fold 4) | Recall (average) |
|---|---|---|---|---|---|---|
| iECO26_1355 | 0.823 | 0.783 | 0.813 | 0.808 | 0.827 | 0.811 |
| iECOK1_1307 | 0.746 | 0.794 | 0.831 | 0.838 | 0.773 | 0.796 |
| iECP_1309 | 0.800 | 0.850 | 0.823 | 0.850 | 0.808 | 0.826 |
| iECS88_1305 | 0.804 | 0.806 | 0.719 | 0.810 | 0.798 | 0.788 |
| iECSE_1348 | 0.835 | 0.835 | 0.829 | 0.815 | 0.842 | 0.831 |
| iECSF_1327 | 0.792 | 0.654 | 0.844 | 0.823 | 0.844 | 0.791 |
| iECSP_1301 | 0.775 | 0.788 | 0.806 | 0.831 | 0.825 | 0.805 |
| iECUMN_1333 | 0.835 | 0.790 | 0.627 | 0.800 | 0.788 | 0.768 |
| iECW_1372 | 0.813 | 0.846 | 0.804 | 0.823 | 0.819 | 0.821 |
| iECs_1301 | 0.794 | 0.783 | 0.813 | 0.792 | 0.808 | 0.798 |
| iEK1008 | 0.854 | 0.818 | 0.813 | 0.885 | 0.786 | 0.831 |
| iEKO11_1354 | 0.810 | 0.852 | 0.823 | 0.815 | 0.792 | 0.818 |
| iETEC_1333 | 0.819 | 0.806 | 0.831 | 0.846 | 0.810 | 0.823 |
| iEcDH1_1363 | 0.798 | 0.794 | 0.777 | 0.800 | 0.840 | 0.802 |
| iEcE24377_1341 | 0.806 | 0.777 | 0.808 | 0.831 | 0.775 | 0.800 |
| iEcHS_1320 | 0.823 | 0.815 | 0.825 | 0.763 | 0.850 | 0.815 |
| iEcSMS35_1347 | 0.802 | 0.756 | 0.833 | 0.794 | 0.823 | 0.802 |
| iEcolC_1368 | 0.833 | 0.773 | 0.804 | 0.852 | 0.852 | 0.823 |
| iG2583_1286 | 0.823 | 0.781 | 0.810 | 0.808 | 0.790 | 0.803 |
| iHN637 | 0.885 | 0.844 | 0.865 | 0.885 | 0.813 | 0.858 |
| iIS312 | 0.771 | 0.708 | 0.698 | 0.792 | 0.698 | 0.733 |
| iIS312_Amastigote | 0.750 | 0.729 | 0.771 | 0.719 | 0.865 | 0.767 |
| iIS312_Epimastigote | 0.740 | 0.833 | 0.750 | 0.729 | 0.771 | 0.765 |
| iIS312_Trypomastigote | 0.729 | 0.729 | 0.740 | 0.781 | 0.708 | 0.738 |
| iIT341 | 0.833 | 0.875 | 0.875 | 0.792 | 0.813 | 0.838 |
| iJB785 | 0.906 | 0.938 | 0.979 | 0.906 | 0.969 | 0.940 |
| iJN1463 | 0.828 | 0.786 | 0.804 | 0.844 | 0.832 | 0.819 |
| iJN678 | 0.771 | 0.948 | 0.885 | 0.813 | 0.865 | 0.856 |
| iJN746 | 0.849 | 0.880 | 0.818 | 0.833 | 0.813 | 0.839 |
| iJO1366 | 0.785 | 0.813 | 0.829 | 0.815 | 0.790 | 0.806 |
| iJR904 | 0.750 | 0.755 | 0.724 | 0.849 | 0.615 | 0.739 |
| iLB1027_lipid | 0.940 | 0.909 | 0.921 | 0.914 | 0.936 | 0.924 |
| iLF82_1304 | 0.792 | 0.698 | 0.775 | 0.838 | 0.842 | 0.789 |
| iLJ478 | 0.865 | 0.938 | 0.729 | 0.823 | 0.823 | 0.835 |
| iML1515 | 0.831 | 0.802 | 0.875 | 0.810 | 0.831 | 0.830 |
| iMM1415 | 0.780 | 0.821 | 0.744 | 0.777 | 0.808 | 0.786 |
| iMM904 | 0.743 | 0.691 | 0.778 | 0.771 | 0.743 | 0.745 |
| iND750 | 0.781 | 0.839 | 0.714 | 0.797 | 0.813 | 0.789 |
| iNF517 | 0.802 | 0.865 | 0.885 | 0.948 | 0.885 | 0.877 |
| iNJ661 | 0.880 | 0.885 | 0.797 | 0.802 | 0.792 | 0.831 |
| iNRG857_1313 | 0.823 | 0.867 | 0.813 | 0.740 | 0.858 | 0.820 |
| iPC815 | 0.797 | 0.776 | 0.755 | 0.797 | 0.792 | 0.783 |
| iRC1080 | 0.857 | 0.865 | 0.898 | 0.862 | 0.865 | 0.869 |

| GEMs | Recall (fold 0) | Recall (fold 1) | Recall (fold 2) | Recall (fold 3) | Recall (fold 4) | Recall (average) |
|---|---|---|---|---|---|---|
| iSB619 | 0.802 | 0.823 | 0.719 | 0.813 | 0.823 | 0.796 |
| iSBO_1134 | 0.796 | 0.792 | 0.825 | 0.788 | 0.792 | 0.798 |
| iSDY_1059 | 0.790 | 0.808 | 0.790 | 0.792 | 0.867 | 0.809 |
| iSFV_1184 | 0.821 | 0.794 | 0.815 | 0.810 | 0.781 | 0.804 |
| iSF_1195 | 0.813 | 0.846 | 0.825 | 0.835 | 0.833 | 0.830 |
| iSFxv_1172 | 0.800 | 0.779 | 0.802 | 0.825 | 0.844 | 0.810 |
| iSSON_1240 | 0.660 | 0.810 | 0.817 | 0.779 | 0.852 | 0.784 |
| iS_1188 | 0.794 | 0.796 | 0.854 | 0.808 | 0.794 | 0.809 |
| iSbBS512_1146 | 0.819 | 0.798 | 0.790 | 0.769 | 0.821 | 0.799 |
| iSynCJ816 | 0.766 | 0.708 | 0.906 | 0.859 | 0.833 | 0.815 |
| iUMN146_1321 | 0.860 | 0.810 | 0.856 | 0.867 | 0.767 | 0.832 |
| iUMNK88_1353 | 0.825 | 0.831 | 0.821 | 0.815 | 0.806 | 0.820 |
| iUTI89_1310 | 0.794 | 0.802 | 0.840 | 0.765 | 0.838 | 0.808 |
| iWFL_1372 | 0.823 | 0.863 | 0.752 | 0.850 | 0.829 | 0.823 |
| iY75_1357 | 0.800 | 0.842 | 0.829 | 0.783 | 0.819 | 0.815 |
| iYL1228 | 0.831 | 0.802 | 0.789 | 0.859 | 0.750 | 0.806 |
| iYO844 | 0.802 | 0.865 | 0.714 | 0.802 | 0.771 | 0.791 |
| iYS1720 | 0.811 | 0.854 | 0.800 | 0.811 | 0.821 | 0.819 |
| iYS854 | 0.653 | 0.816 | 0.792 | 0.750 | 0.715 | 0.745 |
| iZ_1308 | 0.819 | 0.813 | 0.800 | 0.819 | 0.796 | 0.809 |
| ic_1306 | 0.750 | 0.823 | 0.815 | 0.769 | 0.821 | 0.795 |

| GEMs | F1 score (fold 0) | F1 score (fold 1) | F1 score (fold 2) | F1 score (fold 3) | F1 score (fold 4) | F1 score (average) |
|---|---|---|---|---|---|---|
| RECON1 | 0.842 | 0.822 | 0.822 | 0.838 | 0.818 | 0.828 |
| Recon3D | 0.835 | 0.833 | 0.812 | 0.828 | 0.820 | 0.826 |
| STM_v1_0 | 0.811 | 0.813 | 0.810 | 0.820 | 0.806 | 0.812 |
| e_coli_core | 0.519 | 0.737 | 0.600 | 0.625 | 0.800 | 0.656 |
| iAB_RBC_283 | 0.803 | 0.705 | 0.750 | 0.755 | 0.777 | 0.758 |
| iAF1260 | 0.804 | 0.805 | 0.829 | 0.843 | 0.814 | 0.819 |
| iAF1260b | 0.821 | 0.828 | 0.839 | 0.820 | 0.822 | 0.826 |
| iAF692 | 0.844 | 0.846 | 0.842 | 0.800 | 0.767 | 0.820 |
| iAF987 | 0.877 | 0.841 | 0.861 | 0.874 | 0.869 | 0.864 |
| iAM_Pb448 | 0.770 | 0.800 | 0.819 | 0.735 | 0.786 | 0.782 |
| iAM_Pc455 | 0.752 | 0.797 | 0.724 | 0.786 | 0.744 | 0.760 |
| iAM_Pf480 | 0.776 | 0.795 | 0.756 | 0.783 | 0.774 | 0.777 |
| iAM_Pk459 | 0.771 | 0.772 | 0.788 | 0.738 | 0.760 | 0.766 |
| iAM_Pv461 | 0.788 | 0.737 | 0.756 | 0.767 | 0.779 | 0.765 |
| iAPECO1_1312 | 0.798 | 0.802 | 0.764 | 0.836 | 0.814 | 0.803 |
| iAT_PLT_636 | 0.786 | 0.830 | 0.838 | 0.850 | 0.833 | 0.827 |
| iB21_1397 | 0.822 | 0.825 | 0.819 | 0.816 | 0.825 | 0.821 |
| iBWG_1329 | 0.822 | 0.827 | 0.787 | 0.794 | 0.834 | 0.813 |
| iCHOv1 | 0.881 | 0.871 | 0.873 | 0.861 | 0.887 | 0.874 |
| iCHOv1_DG44 | 0.803 | 0.781 | 0.784 | 0.789 | 0.790 | 0.789 |
| iCN718 | 0.805 | 0.847 | 0.813 | 0.767 | 0.840 | 0.814 |
| iCN900 | 0.813 | 0.859 | 0.821 | 0.857 | 0.874 | 0.845 |
| iE2348C_1286 | 0.825 | 0.827 | 0.814 | 0.824 | 0.834 | 0.825 |
| iEC042_1314 | 0.817 | 0.734 | 0.812 | 0.814 | 0.843 | 0.804 |
| iEC1344_C | 0.852 | 0.823 | 0.822 | 0.831 | 0.841 | 0.834 |
| iEC1349_Crooks | 0.827 | 0.810 | 0.833 | 0.817 | 0.846 | 0.827 |
| iEC1356_Bl21DE3 | 0.848 | 0.822 | 0.815 | 0.840 | 0.844 | 0.834 |
| iEC1364_W | 0.863 | 0.828 | 0.821 | 0.846 | 0.855 | 0.843 |
| iEC1368_DH5a | 0.830 | 0.851 | 0.836 | 0.842 | 0.828 | 0.837 |
| iEC1372_W3110 | 0.843 | 0.804 | 0.838 | 0.834 | 0.807 | 0.825 |
| iEC55989_1330 | 0.848 | 0.803 | 0.827 | 0.816 | 0.821 | 0.823 |
| iECABU_c1320 | 0.802 | 0.825 | 0.839 | 0.808 | 0.821 | 0.819 |
| iECBD_1354 | 0.819 | 0.815 | 0.829 | 0.814 | 0.842 | 0.824 |
| iECB_1328 | 0.828 | 0.828 | 0.828 | 0.828 | 0.817 | 0.826 |
| iECDH10B_1368 | 0.804 | 0.799 | 0.825 | 0.821 | 0.856 | 0.821 |
| iECDH1ME8569_1439 | 0.754 | 0.810 | 0.813 | 0.843 | 0.813 | 0.807 |
| iECD_1391 | 0.802 | 0.825 | 0.836 | 0.833 | 0.836 | 0.826 |
| iECED1_1282 | 0.821 | 0.825 | 0.804 | 0.780 | 0.814 | 0.809 |
| iECH74115_1262 | 0.812 | 0.807 | 0.823 | 0.762 | 0.809 | 0.803 |
| iECIAI1_1343 | 0.823 | 0.807 | 0.826 | 0.802 | 0.829 | 0.817 |
| iECIAI39_1322 | 0.807 | 0.796 | 0.814 | 0.811 | 0.800 | 0.805 |
| iECNA114_1301 | 0.829 | 0.843 | 0.801 | 0.819 | 0.813 | 0.821 |
| iECO103_1326 | 0.855 | 0.838 | 0.816 | 0.839 | 0.825 | 0.835 |
| iECO111_1330 | 0.812 | 0.797 | 0.804 | 0.831 | 0.844 | 0.817 |
| iECO26_1355 | 0.828 | 0.805 | 0.819 | 0.815 | 0.839 | 0.821 |

| GEMs | F1 score (fold 0) | F1 score (fold 1) | F1 score (fold 2) | F1 score (fold 3) | F1 score (fold 4) | F1 score (average) |
|---|---|---|---|---|---|---|
| iECOK1_1307 | 0.792 | 0.810 | 0.842 | 0.827 | 0.810 | 0.816 |
| iECP_1309 | 0.828 | 0.846 | 0.818 | 0.831 | 0.821 | 0.829 |
| iECS88_1305 | 0.813 | 0.811 | 0.789 | 0.814 | 0.814 | 0.808 |
| iECSE_1348 | 0.846 | 0.844 | 0.821 | 0.815 | 0.831 | 0.832 |
| iECSF_1327 | 0.805 | 0.748 | 0.839 | 0.821 | 0.824 | 0.807 |
| iECSP_1301 | 0.803 | 0.803 | 0.820 | 0.850 | 0.834 | 0.822 |
| iECUMN_1333 | 0.832 | 0.810 | 0.732 | 0.821 | 0.819 | 0.803 |
| iECW_1372 | 0.824 | 0.854 | 0.817 | 0.831 | 0.847 | 0.834 |
| iECs_1301 | 0.808 | 0.798 | 0.811 | 0.808 | 0.821 | 0.809 |
| iEK1008 | 0.872 | 0.863 | 0.867 | 0.897 | 0.827 | 0.865 |
| iEKO11_1354 | 0.832 | 0.842 | 0.824 | 0.823 | 0.830 | 0.830 |
| iETEC_1333 | 0.816 | 0.810 | 0.823 | 0.826 | 0.826 | 0.820 |
| iEcDH1_1363 | 0.809 | 0.823 | 0.815 | 0.819 | 0.850 | 0.823 |
| iEcE24377_1341 | 0.817 | 0.809 | 0.819 | 0.853 | 0.821 | 0.824 |
| iEcHS_1320 | 0.820 | 0.821 | 0.816 | 0.804 | 0.854 | 0.823 |
| iEcSMS35_1347 | 0.827 | 0.800 | 0.826 | 0.820 | 0.813 | 0.817 |
| iEcolC_1368 | 0.820 | 0.797 | 0.827 | 0.847 | 0.844 | 0.827 |
| iG2583_1286 | 0.831 | 0.799 | 0.810 | 0.822 | 0.817 | 0.816 |
| iHN637 | 0.837 | 0.802 | 0.874 | 0.825 | 0.839 | 0.835 |
| iIS312 | 0.740 | 0.743 | 0.680 | 0.741 | 0.753 | 0.732 |
| iIS312_Amastigote | 0.735 | 0.693 | 0.736 | 0.754 | 0.769 | 0.737 |
| iIS312_Epimastigote | 0.755 | 0.769 | 0.738 | 0.697 | 0.729 | 0.738 |
| iIS312_Trypomastigote | 0.745 | 0.725 | 0.785 | 0.761 | 0.716 | 0.746 |
| iIT341 | 0.784 | 0.820 | 0.789 | 0.768 | 0.772 | 0.787 |
| iJB785 | 0.825 | 0.874 | 0.891 | 0.870 | 0.865 | 0.865 |
| iJN1463 | 0.830 | 0.826 | 0.840 | 0.847 | 0.839 | 0.836 |
| iJN678 | 0.841 | 0.915 | 0.895 | 0.862 | 0.830 | 0.868 |
| iJN746 | 0.847 | 0.864 | 0.858 | 0.853 | 0.832 | 0.851 |
| iJO1366 | 0.822 | 0.820 | 0.818 | 0.820 | 0.815 | 0.819 |
| iJR904 | 0.804 | 0.806 | 0.803 | 0.845 | 0.744 | 0.801 |
| iLB1027_lipid | 0.938 | 0.931 | 0.934 | 0.928 | 0.937 | 0.934 |
| iLF82_1304 | 0.820 | 0.771 | 0.785 | 0.833 | 0.843 | 0.810 |
| iLJ478 | 0.834 | 0.878 | 0.795 | 0.836 | 0.794 | 0.828 |
| iML1515 | 0.826 | 0.811 | 0.844 | 0.827 | 0.830 | 0.828 |
| iMM1415 | 0.805 | 0.815 | 0.778 | 0.801 | 0.812 | 0.802 |
| iMM904 | 0.782 | 0.733 | 0.783 | 0.807 | 0.797 | 0.781 |
| iND750 | 0.798 | 0.801 | 0.761 | 0.799 | 0.825 | 0.797 |
| iNF517 | 0.778 | 0.838 | 0.842 | 0.867 | 0.821 | 0.829 |
| iNJ661 | 0.858 | 0.874 | 0.852 | 0.844 | 0.849 | 0.855 |
| iNRG857_1313 | 0.820 | 0.837 | 0.818 | 0.776 | 0.843 | 0.819 |
| iPC815 | 0.804 | 0.796 | 0.766 | 0.824 | 0.809 | 0.800 |
| iRC1080 | 0.877 | 0.870 | 0.860 | 0.850 | 0.859 | 0.863 |
| iSB619 | 0.824 | 0.814 | 0.798 | 0.830 | 0.845 | 0.822 |
| iSBO_1134 | 0.812 | 0.820 | 0.818 | 0.797 | 0.835 | 0.816 |
| iSDY_1059 | 0.826 | 0.808 | 0.826 | 0.792 | 0.865 | 0.823 |

| GEMs | F1 score (fold 0) | F1 score (fold 1) | F1 score (fold 2) | F1 score (fold 3) | F1 score (fold 4) | F1 score (average) |
|---|---|---|---|---|---|---|
| iSFV_1184 | 0.817 | 0.808 | 0.803 | 0.811 | 0.807 | 0.809 |
| iSF_1195 | 0.819 | 0.807 | 0.820 | 0.833 | 0.823 | 0.820 |
| iSFxv_1172 | 0.802 | 0.803 | 0.808 | 0.799 | 0.831 | 0.808 |
| iSSON_1240 | 0.769 | 0.835 | 0.812 | 0.809 | 0.857 | 0.816 |
| iS_1188 | 0.816 | 0.814 | 0.848 | 0.836 | 0.813 | 0.825 |
| iSbBS512_1146 | 0.811 | 0.800 | 0.814 | 0.795 | 0.821 | 0.808 |
| iSynCJ816 | 0.821 | 0.773 | 0.888 | 0.882 | 0.874 | 0.848 |
| iUMN146_1321 | 0.834 | 0.802 | 0.833 | 0.847 | 0.808 | 0.825 |
| iUMNK88_1353 | 0.818 | 0.830 | 0.812 | 0.835 | 0.824 | 0.824 |
| iUTI89_1310 | 0.821 | 0.826 | 0.834 | 0.792 | 0.836 | 0.822 |
| iWFL_1372 | 0.814 | 0.847 | 0.783 | 0.840 | 0.825 | 0.822 |
| iY75_1357 | 0.827 | 0.830 | 0.822 | 0.809 | 0.826 | 0.823 |
| iYL1228 | 0.860 | 0.845 | 0.815 | 0.876 | 0.810 | 0.841 |
| iYO844 | 0.837 | 0.824 | 0.792 | 0.824 | 0.804 | 0.816 |
| iYS1720 | 0.834 | 0.825 | 0.831 | 0.808 | 0.824 | 0.824 |
| iYS854 | 0.733 | 0.791 | 0.793 | 0.781 | 0.788 | 0.777 |
| iZ_1308 | 0.821 | 0.816 | 0.821 | 0.821 | 0.812 | 0.818 |
| ic_1306 | 0.789 | 0.814 | 0.827 | 0.811 | 0.823 | 0.813 |

| GEMs | Accuracy (fold 0) | Accuracy (fold 1) | Accuracy (fold 2) | Accuracy (fold 3) | Accuracy (fold 4) | Accuracy (average) |
|---|---|---|---|---|---|---|
| RECON1 | 0.844 | 0.819 | 0.822 | 0.839 | 0.818 | 0.828 |
| Recon3D | 0.839 | 0.832 | 0.819 | 0.832 | 0.824 | 0.829 |
| STM_v1_0 | 0.814 | 0.821 | 0.817 | 0.814 | 0.807 | 0.814 |
| e_coli_core | 0.594 | 0.688 | 0.500 | 0.625 | 0.781 | 0.638 |
| iAB_RBC_283 | 0.781 | 0.680 | 0.734 | 0.711 | 0.758 | 0.733 |
| iAF1260 | 0.802 | 0.803 | 0.824 | 0.839 | 0.809 | 0.815 |
| iAF1260b | 0.822 | 0.831 | 0.839 | 0.814 | 0.824 | 0.826 |
| iAF692 | 0.839 | 0.833 | 0.849 | 0.823 | 0.792 | 0.827 |
| iAF987 | 0.878 | 0.831 | 0.859 | 0.875 | 0.865 | 0.861 |
| iAM_Pb448 | 0.781 | 0.807 | 0.815 | 0.745 | 0.794 | 0.789 |
| iAM_Pc455 | 0.779 | 0.805 | 0.766 | 0.784 | 0.753 | 0.777 |
| iAM_Pf480 | 0.776 | 0.781 | 0.755 | 0.763 | 0.768 | 0.769 |
| iAM_Pk459 | 0.758 | 0.781 | 0.781 | 0.745 | 0.755 | 0.764 |
| iAM_Pv461 | 0.786 | 0.740 | 0.750 | 0.771 | 0.779 | 0.765 |
| iAPECO1_1312 | 0.800 | 0.803 | 0.795 | 0.834 | 0.819 | 0.810 |
| iAT_PLT_636 | 0.789 | 0.833 | 0.839 | 0.849 | 0.839 | 0.830 |
| iB21_1397 | 0.829 | 0.832 | 0.823 | 0.821 | 0.834 | 0.828 |
| iBWG_1329 | 0.823 | 0.832 | 0.794 | 0.800 | 0.833 | 0.816 |
| iCHOv1 | 0.882 | 0.875 | 0.875 | 0.865 | 0.887 | 0.877 |
| iCHOv1_DG44 | 0.808 | 0.786 | 0.788 | 0.791 | 0.794 | 0.793 |
| iCN718 | 0.820 | 0.857 | 0.823 | 0.789 | 0.854 | 0.829 |
| iCN900 | 0.823 | 0.854 | 0.820 | 0.857 | 0.870 | 0.845 |
| iE2348C_1286 | 0.829 | 0.826 | 0.813 | 0.826 | 0.835 | 0.826 |
| iEC042_1314 | 0.822 | 0.773 | 0.810 | 0.808 | 0.841 | 0.811 |
| iEC1344_C | 0.855 | 0.826 | 0.817 | 0.830 | 0.842 | 0.834 |
| iEC1349_Crooks | 0.831 | 0.808 | 0.835 | 0.826 | 0.850 | 0.830 |
| iEC1356_Bl21DE3 | 0.850 | 0.828 | 0.820 | 0.838 | 0.844 | 0.836 |
| iEC1364_W | 0.864 | 0.829 | 0.820 | 0.846 | 0.856 | 0.843 |
| iEC1368_DH5a | 0.835 | 0.854 | 0.838 | 0.841 | 0.827 | 0.839 |
| iEC1372_W3110 | 0.844 | 0.813 | 0.836 | 0.833 | 0.816 | 0.828 |
| iEC55989_1330 | 0.848 | 0.810 | 0.834 | 0.815 | 0.825 | 0.826 |
| iECABU_c1320 | 0.803 | 0.825 | 0.845 | 0.810 | 0.823 | 0.821 |
| iECBD_1354 | 0.821 | 0.821 | 0.822 | 0.810 | 0.841 | 0.823 |
| iECB_1328 | 0.832 | 0.833 | 0.829 | 0.832 | 0.813 | 0.828 |
| iECDH10B_1368 | 0.817 | 0.802 | 0.826 | 0.822 | 0.858 | 0.825 |
| iECDH1ME8569_1439 | 0.785 | 0.825 | 0.821 | 0.846 | 0.814 | 0.818 |
| iECD_1391 | 0.798 | 0.828 | 0.840 | 0.831 | 0.838 | 0.827 |
| iECED1_1282 | 0.830 | 0.823 | 0.809 | 0.795 | 0.822 | 0.816 |
| iECH74115_1262 | 0.822 | 0.803 | 0.820 | 0.785 | 0.807 | 0.808 |
| iECIAI1_1343 | 0.828 | 0.803 | 0.830 | 0.803 | 0.830 | 0.819 |
| iECIAI39_1322 | 0.809 | 0.810 | 0.815 | 0.813 | 0.803 | 0.810 |
| iECNA114_1301 | 0.833 | 0.846 | 0.811 | 0.818 | 0.809 | 0.824 |
| iECO103_1326 | 0.855 | 0.840 | 0.814 | 0.841 | 0.832 | 0.836 |
| iECO111_1330 | 0.816 | 0.795 | 0.816 | 0.831 | 0.846 | 0.821 |
| iECO26_1355 | 0.829 | 0.810 | 0.821 | 0.817 | 0.842 | 0.824 |

| GEMs | Accuracy (fold 0) | Accuracy (fold 1) | Accuracy (fold 2) | Accuracy (fold 3) | Accuracy (fold 4) | Accuracy (average) |
|---|---|---|---|---|---|---|
| iECOK1_1307 | 0.804 | 0.814 | 0.844 | 0.825 | 0.819 | 0.821 |
| iECP_1309 | 0.834 | 0.845 | 0.817 | 0.827 | 0.824 | 0.829 |
| iECS88_1305 | 0.816 | 0.813 | 0.807 | 0.815 | 0.818 | 0.814 |
| iECSE_1348 | 0.848 | 0.846 | 0.820 | 0.815 | 0.829 | 0.831 |
| iECSF_1327 | 0.808 | 0.779 | 0.839 | 0.821 | 0.820 | 0.813 |
| iECSP_1301 | 0.809 | 0.807 | 0.823 | 0.853 | 0.835 | 0.826 |
| iECUMN_1333 | 0.831 | 0.815 | 0.771 | 0.826 | 0.826 | 0.814 |
| iECW_1372 | 0.826 | 0.855 | 0.820 | 0.832 | 0.852 | 0.837 |
| iECs_1301 | 0.811 | 0.802 | 0.810 | 0.811 | 0.824 | 0.812 |
| iEK1008 | 0.875 | 0.870 | 0.875 | 0.898 | 0.836 | 0.871 |
| iEKO11_1354 | 0.836 | 0.841 | 0.824 | 0.825 | 0.838 | 0.833 |
| iETEC_1333 | 0.816 | 0.811 | 0.821 | 0.822 | 0.829 | 0.820 |
| iEcDH1_1363 | 0.811 | 0.829 | 0.824 | 0.823 | 0.852 | 0.828 |
| iEcE24377_1341 | 0.820 | 0.817 | 0.822 | 0.857 | 0.831 | 0.829 |
| iEcHS_1320 | 0.820 | 0.823 | 0.815 | 0.814 | 0.854 | 0.825 |
| iEcSMS35_1347 | 0.832 | 0.810 | 0.825 | 0.826 | 0.810 | 0.821 |
| iEcolC_1368 | 0.817 | 0.803 | 0.831 | 0.846 | 0.843 | 0.828 |
| iG2583_1286 | 0.832 | 0.803 | 0.810 | 0.825 | 0.823 | 0.819 |
| iHN637 | 0.828 | 0.792 | 0.875 | 0.813 | 0.844 | 0.830 |
| iIS312 | 0.729 | 0.755 | 0.672 | 0.724 | 0.771 | 0.730 |
| iIS312_Amastigote | 0.729 | 0.677 | 0.724 | 0.766 | 0.740 | 0.727 |
| iIS312_Epimastigote | 0.760 | 0.750 | 0.734 | 0.682 | 0.714 | 0.728 |
| iIS312_Trypomastigote | 0.750 | 0.724 | 0.797 | 0.755 | 0.719 | 0.749 |
| iIT341 | 0.771 | 0.807 | 0.766 | 0.760 | 0.760 | 0.773 |
| iJB785 | 0.807 | 0.865 | 0.880 | 0.865 | 0.849 | 0.853 |
| iJN1463 | 0.831 | 0.834 | 0.846 | 0.847 | 0.840 | 0.840 |
| iJN678 | 0.854 | 0.911 | 0.896 | 0.870 | 0.823 | 0.871 |
| iJN746 | 0.846 | 0.862 | 0.865 | 0.857 | 0.836 | 0.853 |
| iJO1366 | 0.830 | 0.822 | 0.816 | 0.821 | 0.821 | 0.822 |
| iJR904 | 0.818 | 0.818 | 0.823 | 0.844 | 0.789 | 0.818 |
| iLB1027_lipid | 0.938 | 0.933 | 0.935 | 0.929 | 0.938 | 0.935 |
| iLF82_1304 | 0.826 | 0.793 | 0.788 | 0.832 | 0.843 | 0.816 |
| iLJ478 | 0.828 | 0.870 | 0.813 | 0.839 | 0.786 | 0.827 |
| iML1515 | 0.825 | 0.813 | 0.839 | 0.830 | 0.830 | 0.827 |
| iMM1415 | 0.811 | 0.814 | 0.788 | 0.807 | 0.813 | 0.806 |
| iMM904 | 0.793 | 0.748 | 0.785 | 0.816 | 0.811 | 0.791 |
| iND750 | 0.802 | 0.792 | 0.776 | 0.799 | 0.828 | 0.799 |
| iNF517 | 0.771 | 0.833 | 0.833 | 0.854 | 0.807 | 0.820 |
| iNJ661 | 0.854 | 0.872 | 0.862 | 0.852 | 0.859 | 0.860 |
| iNRG857_1313 | 0.819 | 0.831 | 0.819 | 0.786 | 0.840 | 0.819 |
| iPC815 | 0.806 | 0.801 | 0.770 | 0.829 | 0.813 | 0.804 |
| iRC1080 | 0.880 | 0.871 | 0.854 | 0.848 | 0.858 | 0.862 |
| iSB619 | 0.828 | 0.813 | 0.818 | 0.833 | 0.849 | 0.828 |
| iSBO_1134 | 0.816 | 0.826 | 0.817 | 0.799 | 0.844 | 0.820 |
| iSDY_1059 | 0.833 | 0.808 | 0.833 | 0.793 | 0.865 | 0.826 |

| GEMs | Accuracy (fold 0) | Accuracy (fold 1) | Accuracy (fold 2) | Accuracy (fold 3) | Accuracy (fold 4) | Accuracy (average) |
|---|---|---|---|---|---|---|
| iSFV_1184 | 0.817 | 0.811 | 0.800 | 0.811 | 0.814 | 0.811 |
| iSF_1195 | 0.821 | 0.798 | 0.819 | 0.832 | 0.821 | 0.818 |
| iSFxv_1172 | 0.802 | 0.808 | 0.809 | 0.793 | 0.828 | 0.808 |
| iSSON_1240 | 0.802 | 0.840 | 0.810 | 0.816 | 0.857 | 0.825 |
| iS_1188 | 0.821 | 0.818 | 0.847 | 0.842 | 0.818 | 0.829 |
| iSbBS512_1146 | 0.809 | 0.801 | 0.820 | 0.802 | 0.821 | 0.811 |
| iSynCJ816 | 0.833 | 0.792 | 0.885 | 0.885 | 0.880 | 0.855 |
| iUMN146_1321 | 0.829 | 0.800 | 0.828 | 0.844 | 0.818 | 0.824 |
| iUMNK88_1353 | 0.817 | 0.830 | 0.810 | 0.840 | 0.828 | 0.825 |
| iUTI89_1310 | 0.827 | 0.831 | 0.832 | 0.799 | 0.835 | 0.825 |
| iWFL_1372 | 0.811 | 0.845 | 0.792 | 0.839 | 0.824 | 0.822 |
| iY75_1357 | 0.832 | 0.827 | 0.821 | 0.815 | 0.828 | 0.825 |
| iYL1228 | 0.865 | 0.853 | 0.820 | 0.879 | 0.824 | 0.848 |
| iYO844 | 0.844 | 0.815 | 0.813 | 0.828 | 0.813 | 0.822 |
| iYS1720 | 0.839 | 0.818 | 0.837 | 0.807 | 0.825 | 0.825 |
| iYS854 | 0.762 | 0.785 | 0.793 | 0.790 | 0.807 | 0.788 |
| iZ_1308 | 0.822 | 0.817 | 0.825 | 0.822 | 0.816 | 0.820 |
| ic_1306 | 0.799 | 0.811 | 0.829 | 0.821 | 0.824 | 0.817 |

| GEMs | Precision (fold 0) | Precision (fold 1) | Precision (fold 2) | Precision (fold 3) | Precision (fold 4) | Precision (average) |
|---|---|---|---|---|---|---|
| RECON1 | 0.853 | 0.808 | 0.825 | 0.845 | 0.815 | 0.829 |
| Recon3D | 0.855 | 0.830 | 0.842 | 0.848 | 0.836 | 0.842 |
| STM_v1_0 | 0.821 | 0.850 | 0.841 | 0.792 | 0.812 | 0.823 |
| e_coli_core | 0.636 | 0.636 | 0.500 | 0.625 | 0.737 | 0.627 |
| iAB_RBC_283 | 0.731 | 0.653 | 0.708 | 0.655 | 0.720 | 0.694 |
| iAF1260 | 0.797 | 0.798 | 0.807 | 0.820 | 0.793 | 0.803 |
| iAF1260b | 0.824 | 0.840 | 0.837 | 0.793 | 0.834 | 0.825 |
| iAF692 | 0.816 | 0.786 | 0.885 | 0.919 | 0.868 | 0.855 |
| iAF987 | 0.884 | 0.793 | 0.852 | 0.879 | 0.840 | 0.849 |
| iAM_Pb448 | 0.810 | 0.831 | 0.801 | 0.764 | 0.819 | 0.805 |
| iAM_Pc455 | 0.854 | 0.831 | 0.881 | 0.779 | 0.771 | 0.823 |
| iAM_Pf480 | 0.776 | 0.748 | 0.753 | 0.722 | 0.756 | 0.751 |
| iAM_Pk459 | 0.730 | 0.807 | 0.765 | 0.758 | 0.745 | 0.761 |
| iAM_Pv461 | 0.784 | 0.745 | 0.738 | 0.780 | 0.777 | 0.765 |
| iAPECO1_1312 | 0.806 | 0.806 | 0.901 | 0.827 | 0.836 | 0.835 |
| iAT_PLT_636 | 0.797 | 0.848 | 0.842 | 0.845 | 0.861 | 0.839 |
| iB21_1397 | 0.857 | 0.863 | 0.837 | 0.839 | 0.872 | 0.854 |
| iBWG_1329 | 0.827 | 0.855 | 0.815 | 0.817 | 0.832 | 0.829 |
| iCHOv1 | 0.888 | 0.906 | 0.887 | 0.883 | 0.887 | 0.890 |
| iCHOv1_DG44 | 0.824 | 0.799 | 0.799 | 0.798 | 0.805 | 0.805 |
| iCN718 | 0.882 | 0.910 | 0.860 | 0.858 | 0.930 | 0.888 |
| iCN900 | 0.860 | 0.833 | 0.819 | 0.855 | 0.845 | 0.842 |
| iE2348C_1286 | 0.845 | 0.823 | 0.806 | 0.835 | 0.843 | 0.830 |
| iEC042_1314 | 0.841 | 0.885 | 0.804 | 0.790 | 0.829 | 0.830 |
| iEC1344_C | 0.871 | 0.839 | 0.798 | 0.827 | 0.846 | 0.836 |
| iEC1349_Crooks | 0.847 | 0.802 | 0.845 | 0.863 | 0.867 | 0.845 |
| iEC1356_Bl21DE3 | 0.859 | 0.854 | 0.836 | 0.828 | 0.842 | 0.844 |
| iEC1364_W | 0.864 | 0.832 | 0.815 | 0.846 | 0.862 | 0.844 |
| iEC1368_DH5a | 0.859 | 0.868 | 0.845 | 0.834 | 0.823 | 0.846 |
| iEC1372_W3110 | 0.845 | 0.842 | 0.829 | 0.831 | 0.845 | 0.838 |
| iEC55989_1330 | 0.848 | 0.834 | 0.867 | 0.809 | 0.842 | 0.840 |
| iECABU_c1320 | 0.808 | 0.825 | 0.874 | 0.817 | 0.830 | 0.831 |
| iECBD_1354 | 0.828 | 0.844 | 0.797 | 0.800 | 0.836 | 0.821 |
| iECB_1328 | 0.851 | 0.856 | 0.835 | 0.851 | 0.799 | 0.838 |
| iECDH10B_1368 | 0.864 | 0.813 | 0.831 | 0.825 | 0.869 | 0.840 |
| iECDH1ME8569_1439 | 0.885 | 0.886 | 0.850 | 0.859 | 0.814 | 0.859 |
| iECD_1391 | 0.787 | 0.840 | 0.856 | 0.824 | 0.843 | 0.830 |
| iECED1_1282 | 0.868 | 0.815 | 0.826 | 0.841 | 0.852 | 0.840 |
| iECH74115_1262 | 0.860 | 0.790 | 0.810 | 0.855 | 0.803 | 0.824 |
| iECIAI1_1343 | 0.848 | 0.792 | 0.848 | 0.808 | 0.837 | 0.826 |
| iECIAI39_1322 | 0.818 | 0.862 | 0.817 | 0.818 | 0.813 | 0.826 |
| iECNA114_1301 | 0.849 | 0.861 | 0.848 | 0.814 | 0.798 | 0.834 |
| iECO103_1326 | 0.854 | 0.848 | 0.804 | 0.849 | 0.862 | 0.843 |
| iECO111_1330 | 0.829 | 0.789 | 0.858 | 0.833 | 0.856 | 0.833 |
| iECO26_1355 | 0.833 | 0.828 | 0.826 | 0.822 | 0.852 | 0.832 |

| GEMs | Precision (fold 0) | Precision (fold 1) | Precision (fold 2) | Precision (fold 3) | Precision (fold 4) | Precision (average) |
|---|---|---|---|---|---|---|
| iECOK1_1307 | 0.844 | 0.826 | 0.853 | 0.817 | 0.851 | 0.838 |
| iECP_1309 | 0.859 | 0.841 | 0.813 | 0.813 | 0.834 | 0.832 |
| iECS88_1305 | 0.823 | 0.816 | 0.873 | 0.817 | 0.831 | 0.832 |
| iECSE_1348 | 0.857 | 0.853 | 0.814 | 0.815 | 0.821 | 0.832 |
| iECSF_1327 | 0.819 | 0.872 | 0.835 | 0.820 | 0.805 | 0.830 |
| iECSP_1301 | 0.832 | 0.820 | 0.834 | 0.869 | 0.843 | 0.840 |
| iECUMN_1333 | 0.829 | 0.831 | 0.880 | 0.844 | 0.853 | 0.847 |
| iECW_1372 | 0.835 | 0.862 | 0.830 | 0.839 | 0.877 | 0.849 |
| iECs_1301 | 0.823 | 0.814 | 0.809 | 0.824 | 0.834 | 0.821 |
| iEK1008 | 0.891 | 0.913 | 0.929 | 0.909 | 0.873 | 0.903 |
| iEKO11_1354 | 0.855 | 0.833 | 0.825 | 0.832 | 0.872 | 0.843 |
| iETEC_1333 | 0.814 | 0.815 | 0.814 | 0.807 | 0.842 | 0.818 |
| iEcDH1_1363 | 0.820 | 0.854 | 0.857 | 0.838 | 0.861 | 0.846 |
| iEcE24377_1341 | 0.829 | 0.844 | 0.831 | 0.877 | 0.873 | 0.851 |
| iEcHS_1320 | 0.818 | 0.828 | 0.808 | 0.849 | 0.857 | 0.832 |
| iEcSMS35_1347 | 0.854 | 0.848 | 0.820 | 0.849 | 0.803 | 0.835 |
| iEcolC_1368 | 0.806 | 0.823 | 0.850 | 0.842 | 0.836 | 0.831 |
| iG2583_1286 | 0.839 | 0.817 | 0.810 | 0.836 | 0.846 | 0.830 |
| iHN637 | 0.794 | 0.764 | 0.883 | 0.773 | 0.867 | 0.816 |
| iIS312 | 0.712 | 0.782 | 0.663 | 0.697 | 0.817 | 0.734 |
| iIS312_Amastigote | 0.720 | 0.660 | 0.705 | 0.793 | 0.692 | 0.714 |
| iIS312_Epimastigote | 0.772 | 0.714 | 0.727 | 0.667 | 0.692 | 0.714 |
| iIS312_Trypomastigote | 0.761 | 0.722 | 0.835 | 0.743 | 0.723 | 0.757 |
| iIT341 | 0.741 | 0.771 | 0.718 | 0.745 | 0.736 | 0.742 |
| iJB785 | 0.757 | 0.818 | 0.817 | 0.837 | 0.782 | 0.802 |
| iJN1463 | 0.832 | 0.869 | 0.879 | 0.850 | 0.846 | 0.855 |
| iJN678 | 0.925 | 0.883 | 0.904 | 0.918 | 0.798 | 0.886 |
| iJN746 | 0.845 | 0.849 | 0.902 | 0.874 | 0.852 | 0.865 |
| iJO1366 | 0.863 | 0.828 | 0.807 | 0.825 | 0.842 | 0.833 |
| iJR904 | 0.867 | 0.863 | 0.903 | 0.840 | 0.944 | 0.883 |
| iLB1027_lipid | 0.937 | 0.955 | 0.948 | 0.943 | 0.939 | 0.944 |
| iLF82_1304 | 0.850 | 0.861 | 0.795 | 0.829 | 0.843 | 0.836 |
| iLJ478 | 0.806 | 0.826 | 0.875 | 0.849 | 0.767 | 0.825 |
| iML1515 | 0.821 | 0.819 | 0.816 | 0.844 | 0.830 | 0.826 |
| iMM1415 | 0.832 | 0.809 | 0.816 | 0.826 | 0.815 | 0.820 |
| iMM904 | 0.826 | 0.780 | 0.789 | 0.847 | 0.859 | 0.820 |
| iND750 | 0.815 | 0.767 | 0.815 | 0.801 | 0.839 | 0.807 |
| iNF517 | 0.755 | 0.814 | 0.802 | 0.798 | 0.766 | 0.787 |
| iNJ661 | 0.837 | 0.863 | 0.916 | 0.890 | 0.916 | 0.884 |
| iNRG857_1313 | 0.816 | 0.809 | 0.823 | 0.816 | 0.827 | 0.818 |
| iPC815 | 0.812 | 0.816 | 0.777 | 0.852 | 0.826 | 0.817 |
| iRC1080 | 0.899 | 0.876 | 0.825 | 0.838 | 0.853 | 0.858 |
| iSB619 | 0.846 | 0.806 | 0.896 | 0.848 | 0.868 | 0.853 |
| iSBO_1134 | 0.829 | 0.850 | 0.811 | 0.806 | 0.884 | 0.836 |
| iSDY_1059 | 0.865 | 0.808 | 0.865 | 0.793 | 0.863 | 0.839 |

| GEMs | Precision (fold 0) | Precision (fold 1) | Precision (fold 2) | Precision (fold 3) | Precision (fold 4) | Precision (average) |
|---|---|---|---|---|---|---|
| iSFV_1184 | 0.814 | 0.823 | 0.791 | 0.812 | 0.835 | 0.815 |
| iSF_1195 | 0.826 | 0.772 | 0.815 | 0.830 | 0.813 | 0.811 |
| iSFxv_1172 | 0.803 | 0.827 | 0.814 | 0.775 | 0.818 | 0.808 |
| iSSON_1240 | 0.922 | 0.861 | 0.807 | 0.840 | 0.861 | 0.858 |
| iS_1188 | 0.839 | 0.832 | 0.842 | 0.866 | 0.834 | 0.843 |
| iSbBS512_1146 | 0.804 | 0.803 | 0.840 | 0.824 | 0.821 | 0.818 |
| iSynCJ816 | 0.886 | 0.850 | 0.870 | 0.907 | 0.920 | 0.886 |
| iUMN146_1321 | 0.810 | 0.794 | 0.811 | 0.829 | 0.854 | 0.819 |
| iUMNK88_1353 | 0.811 | 0.830 | 0.804 | 0.857 | 0.843 | 0.829 |
| iUTI89_1310 | 0.850 | 0.852 | 0.828 | 0.821 | 0.834 | 0.837 |
| iWFL_1372 | 0.804 | 0.833 | 0.817 | 0.831 | 0.821 | 0.821 |
| iY75_1357 | 0.855 | 0.818 | 0.816 | 0.836 | 0.834 | 0.832 |
| iYL1228 | 0.891 | 0.893 | 0.842 | 0.894 | 0.881 | 0.880 |
| iYO844 | 0.875 | 0.787 | 0.890 | 0.846 | 0.841 | 0.848 |
| iYS1720 | 0.858 | 0.797 | 0.863 | 0.805 | 0.827 | 0.830 |
| iYS854 | 0.836 | 0.768 | 0.794 | 0.815 | 0.877 | 0.818 |
| iZ_1308 | 0.824 | 0.819 | 0.842 | 0.824 | 0.829 | 0.828 |
| ic_1306 | 0.831 | 0.804 | 0.839 | 0.858 | 0.826 | 0.832 |

**Supplementary Table S5: The detailed results of recovery rate performances of GHCN-SE on 108 BiGG GEMs.**

| GEMs | Top 25 (fold 0) | Top 25 (fold 1) | Top 25 (fold 2) | Top 25 (fold 3) | Top 25 (fold 4) | Top 25 (average) |
|---|---|---|---|---|---|---|
| e_coli_core | 0.160 | 0.150 | 0.120 | 0.120 | 0.120 | 0.134 |
| iAB_RBC_283 | 0.115 | 0.160 | 0.160 | 0.160 | 0.160 | 0.151 |
| iAF1260 | 0.120 | 0.120 | 0.085 | 0.160 | 0.120 | 0.121 |
| iAF1260b | 0.200 | 0.200 | 0.225 | 0.200 | 0.240 | 0.213 |
| iAF692 | 0.160 | 0.160 | 0.160 | 0.120 | 0.170 | 0.154 |
| iAF987 | 0.120 | 0.070 | 0.080 | 0.080 | 0.120 | 0.094 |
| iAM_Pb448 | 0.160 | 0.160 | 0.100 | 0.120 | 0.120 | 0.132 |
| iAM_Pc455 | 0.205 | 0.200 | 0.160 | 0.200 | 0.160 | 0.185 |
| iAM_Pf480 | 0.135 | 0.120 | 0.120 | 0.160 | 0.120 | 0.131 |
| iAM_Pk459 | 0.160 | 0.200 | 0.200 | 0.130 | 0.160 | 0.170 |
| iAM_Pv461 | 0.120 | 0.110 | 0.120 | 0.120 | 0.160 | 0.126 |
| iAPECO1_1312 | 0.160 | 0.160 | 0.185 | 0.120 | 0.120 | 0.149 |
| iAT_PLT_636 | 0.225 | 0.200 | 0.200 | 0.200 | 0.200 | 0.205 |
| iB21_1397 | 0.080 | 0.120 | 0.120 | 0.070 | 0.120 | 0.102 |
| iBWG_1329 | 0.200 | 0.200 | 0.200 | 0.170 | 0.200 | 0.194 |
| ic_1306 | 0.165 | 0.200 | 0.160 | 0.200 | 0.200 | 0.185 |
| iCHOv1 | 0.240 | 0.240 | 0.215 | 0.200 | 0.240 | 0.227 |
| iCHOv1_DG44 | 0.140 | 0.160 | 0.200 | 0.160 | 0.160 | 0.164 |
| iCN718 | 0.200 | 0.205 | 0.240 | 0.200 | 0.200 | 0.209 |
| iCN900 | 0.160 | 0.160 | 0.200 | 0.160 | 0.145 | 0.165 |
| iE2348C_1286 | 0.160 | 0.160 | 0.200 | 0.165 | 0.200 | 0.177 |
| iEC042_1314 | 0.160 | 0.145 | 0.160 | 0.200 | 0.160 | 0.165 |
| iEC1344_C | 0.160 | 0.205 | 0.160 | 0.160 | 0.160 | 0.169 |
| iEC1349_Crooks | 0.200 | 0.175 | 0.200 | 0.240 | 0.240 | 0.211 |
| iEC1356_Bl21DE3 | 0.160 | 0.120 | 0.160 | 0.150 | 0.160 | 0.150 |
| iEC1364_W | 0.200 | 0.160 | 0.170 | 0.160 | 0.200 | 0.178 |
| iEC1368_DH5a | 0.155 | 0.160 | 0.160 | 0.160 | 0.160 | 0.159 |
| iEC1372_W3110 | 0.120 | 0.160 | 0.120 | 0.185 | 0.160 | 0.149 |
| iEC55989_1330 | 0.170 | 0.160 | 0.160 | 0.120 | 0.160 | 0.154 |
| iECABU_c1320 | 0.165 | 0.160 | 0.160 | 0.160 | 0.200 | 0.169 |
| iECB_1328 | 0.160 | 0.160 | 0.160 | 0.120 | 0.165 | 0.153 |
| iECBD_1354 | 0.200 | 0.175 | 0.200 | 0.200 | 0.160 | 0.187 |
| iECD_1391 | 0.200 | 0.180 | 0.200 | 0.200 | 0.240 | 0.204 |
| iEcDH1_1363 | 0.110 | 0.120 | 0.120 | 0.120 | 0.120 | 0.118 |
| iECDH10B_1368 | 0.200 | 0.170 | 0.200 | 0.200 | 0.240 | 0.202 |
| iECDH1ME8569_1439 | 0.160 | 0.095 | 0.160 | 0.120 | 0.120 | 0.131 |
| iEcE24377_1341 | 0.120 | 0.090 | 0.120 | 0.120 | 0.120 | 0.114 |
| iECED1_1282 | 0.120 | 0.085 | 0.160 | 0.120 | 0.120 | 0.121 |
| iECH74115_1262 | 0.160 | 0.145 | 0.160 | 0.120 | 0.120 | 0.141 |
| iEcHS_1320 | 0.200 | 0.200 | 0.200 | 0.170 | 0.200 | 0.194 |
| iECIAI1_1343 | 0.160 | 0.120 | 0.160 | 0.160 | 0.160 | 0.152 |
| iECIAI39_1322 | 0.160 | 0.160 | 0.200 | 0.160 | 0.140 | 0.164 |

| GEMs | Top 25 (fold 0) | Top 25 (fold 1) | Top 25 (fold 2) | Top 25 (fold 3) | Top 25 (fold 4) | Top 25 (average) |
|---|---|---|---|---|---|---|
| iECNA114_1301 | 0.200 | 0.175 | 0.200 | 0.200 | 0.200 | 0.195 |
| iECO103_1326 | 0.235 | 0.160 | 0.200 | 0.200 | 0.200 | 0.199 |
| iECO111_1330 | 0.200 | 0.160 | 0.200 | 0.160 | 0.200 | 0.184 |
| iECO26_1355 | 0.200 | 0.200 | 0.155 | 0.200 | 0.200 | 0.191 |
| iECOK1_1307 | 0.240 | 0.200 | 0.240 | 0.200 | 0.240 | 0.224 |
| iEcolC_1368 | 0.120 | 0.080 | 0.040 | 0.040 | 0.120 | 0.080 |
| iECP_1309 | 0.120 | 0.120 | 0.120 | 0.120 | 0.120 | 0.120 |
| iECs_1301 | 0.125 | 0.120 | 0.160 | 0.120 | 0.120 | 0.129 |
| iECS88_1305 | 0.120 | 0.160 | 0.155 | 0.120 | 0.120 | 0.135 |
| iECSE_1348 | 0.160 | 0.160 | 0.160 | 0.160 | 0.170 | 0.162 |
| iECSF_1327 | 0.080 | 0.120 | 0.080 | 0.080 | 0.120 | 0.096 |
| iEcSMS35_1347 | 0.160 | 0.120 | 0.160 | 0.120 | 0.155 | 0.143 |
| iECSP_1301 | 0.200 | 0.200 | 0.225 | 0.160 | 0.160 | 0.189 |
| iECUMN_1333 | 0.200 | 0.200 | 0.200 | 0.200 | 0.200 | 0.200 |
| iECW_1372 | 0.140 | 0.160 | 0.160 | 0.160 | 0.160 | 0.156 |
| iEK1008 | 0.200 | 0.200 | 0.230 | 0.200 | 0.160 | 0.198 |
| iEKO11_1354 | 0.160 | 0.200 | 0.200 | 0.210 | 0.160 | 0.186 |
| iETEC_1333 | 0.200 | 0.165 | 0.200 | 0.200 | 0.160 | 0.185 |
| iG2583_1286 | 0.160 | 0.120 | 0.115 | 0.160 | 0.160 | 0.143 |
| iHN637 | 0.160 | 0.160 | 0.160 | 0.160 | 0.210 | 0.170 |
| iIS312 | 0.200 | 0.200 | 0.200 | 0.160 | 0.165 | 0.185 |
| iIS312_Amastigote | 0.080 | 0.120 | 0.080 | 0.120 | 0.100 | 0.100 |
| iIS312_Epimastigote | 0.160 | 0.120 | 0.135 | 0.120 | 0.120 | 0.131 |
| iIS312_Trypomastigote | 0.160 | 0.120 | 0.160 | 0.120 | 0.160 | 0.144 |
| iIT341 | 0.180 | 0.200 | 0.160 | 0.160 | 0.200 | 0.180 |
| iJB785 | 0.160 | 0.160 | 0.160 | 0.150 | 0.120 | 0.150 |
| iJN1463 | 0.200 | 0.200 | 0.175 | 0.240 | 0.200 | 0.203 |
| iJN678 | 0.200 | 0.200 | 0.200 | 0.160 | 0.205 | 0.193 |
| iJN746 | 0.160 | 0.160 | 0.160 | 0.115 | 0.160 | 0.151 |
| iJO1366 | 0.120 | 0.120 | 0.120 | 0.155 | 0.080 | 0.119 |
| iJR904 | 0.240 | 0.170 | 0.200 | 0.200 | 0.200 | 0.202 |
| iLB1027_lipid | 0.080 | 0.120 | 0.105 | 0.080 | 0.120 | 0.101 |
| iLF82_1304 | 0.080 | 0.080 | 0.120 | 0.065 | 0.120 | 0.093 |
| iLJ478 | 0.200 | 0.230 | 0.200 | 0.200 | 0.160 | 0.198 |
| iML1515 | 0.160 | 0.200 | 0.175 | 0.160 | 0.200 | 0.179 |
| iMM1415 | 0.080 | 0.080 | 0.080 | 0.080 | 0.100 | 0.084 |
| iMM904 | 0.110 | 0.160 | 0.160 | 0.120 | 0.160 | 0.142 |
| iND750 | 0.180 | 0.120 | 0.120 | 0.120 | 0.160 | 0.140 |
| iNF517 | 0.160 | 0.160 | 0.200 | 0.200 | 0.180 | 0.180 |
| iNJ661 | 0.200 | 0.160 | 0.175 | 0.200 | 0.160 | 0.179 |
| iNRG857_1313 | 0.140 | 0.160 | 0.160 | 0.160 | 0.160 | 0.156 |
| iPC815 | 0.080 | 0.080 | 0.080 | 0.100 | 0.080 | 0.084 |
| iRC1080 | 0.240 | 0.230 | 0.200 | 0.200 | 0.200 | 0.214 |
| iS_1188 | 0.160 | 0.160 | 0.200 | 0.200 | 0.195 | 0.183 |
| iSB619 | 0.200 | 0.210 | 0.160 | 0.200 | 0.160 | 0.186 |

| GEMs | Top 25 (fold 0) | Top 25 (fold 1) | Top 25 (fold 2) | Top 25 (fold 3) | Top 25 (fold 4) | Top 25 (average) |
|---|---|---|---|---|---|---|
| iSbBS512_1146 | 0.200 | 0.155 | 0.200 | 0.200 | 0.200 | 0.191 |
| iSBO_1134 | 0.160 | 0.200 | 0.200 | 0.200 | 0.200 | 0.192 |
| iSDY_1059 | 0.200 | 0.200 | 0.230 | 0.240 | 0.200 | 0.214 |
| iSF_1195 | 0.200 | 0.240 | 0.180 | 0.200 | 0.200 | 0.204 |
| iSFV_1184 | 0.160 | 0.160 | 0.160 | 0.155 | 0.160 | 0.159 |
| iSFxv_1172 | 0.120 | 0.120 | 0.160 | 0.125 | 0.120 | 0.129 |
| iSSON_1240 | 0.200 | 0.200 | 0.230 | 0.240 | 0.200 | 0.214 |
| iSynCJ816 | 0.120 | 0.120 | 0.080 | 0.110 | 0.080 | 0.102 |
| iUMN146_1321 | 0.200 | 0.160 | 0.160 | 0.200 | 0.230 | 0.190 |
| iUMNK88_1353 | 0.160 | 0.225 | 0.160 | 0.200 | 0.200 | 0.189 |
| iUTI89_1310 | 0.115 | 0.120 | 0.120 | 0.080 | 0.120 | 0.111 |
| iWFL_1372 | 0.160 | 0.150 | 0.200 | 0.200 | 0.200 | 0.182 |
| iY75_1357 | 0.160 | 0.160 | 0.160 | 0.160 | 0.195 | 0.167 |
| iYL1228 | 0.145 | 0.120 | 0.120 | 0.120 | 0.160 | 0.133 |
| iYO844 | 0.080 | 0.070 | 0.120 | 0.120 | 0.120 | 0.102 |
| iYS1720 | 0.160 | 0.160 | 0.120 | 0.160 | 0.125 | 0.145 |
| iYS854 | 0.200 | 0.200 | 0.190 | 0.200 | 0.200 | 0.198 |
| iZ_1308 | 0.120 | 0.120 | 0.160 | 0.145 | 0.120 | 0.133 |
| RECON1 | 0.165 | 0.120 | 0.120 | 0.160 | 0.120 | 0.137 |
| Recon3D | 0.160 | 0.160 | 0.160 | 0.175 | 0.200 | 0.171 |
| STM_v1_0 | 0.120 | 0.120 | 0.120 | 0.160 | 0.090 | 0.122 |

| GEMs | Top 50 (fold 0) | Top 50 (fold 1) | Top 50 (fold 2) | Top 50 (fold 3) | Top 50 (fold 4) | Top 50 (average) |
|---|---|---|---|---|---|---|
| e_coli_core | 0.180 | 0.240 | 0.220 | 0.220 | 0.220 | 0.216 |
| iAB_RBC_283 | 0.160 | 0.160 | 0.100 | 0.090 | 0.100 | 0.122 |
| iAF1260 | 0.200 | 0.160 | 0.200 | 0.160 | 0.205 | 0.185 |
| iAF1260b | 0.120 | 0.180 | 0.180 | 0.130 | 0.180 | 0.158 |
| iAF692 | 0.060 | 0.120 | 0.080 | 0.095 | 0.120 | 0.095 |
| iAF987 | 0.180 | 0.180 | 0.180 | 0.190 | 0.140 | 0.174 |
| iAM_Pb448 | 0.080 | 0.080 | 0.060 | 0.065 | 0.100 | 0.077 |
| iAM_Pc455 | 0.160 | 0.120 | 0.160 | 0.120 | 0.130 | 0.138 |
| iAM_Pf480 | 0.080 | 0.100 | 0.080 | 0.060 | 0.065 | 0.077 |
| iAM_Pk459 | 0.080 | 0.140 | 0.100 | 0.140 | 0.090 | 0.110 |
| iAM_Pv461 | 0.100 | 0.080 | 0.100 | 0.120 | 0.125 | 0.105 |
| iAPECO1_1312 | 0.180 | 0.180 | 0.180 | 0.150 | 0.180 | 0.174 |
| iAT_PLT_636 | 0.125 | 0.100 | 0.100 | 0.140 | 0.120 | 0.117 |
| iB21_1397 | 0.185 | 0.140 | 0.140 | 0.140 | 0.180 | 0.157 |
| iBWG_1329 | 0.140 | 0.120 | 0.120 | 0.160 | 0.125 | 0.133 |
| ic_1306 | 0.180 | 0.205 | 0.160 | 0.180 | 0.140 | 0.173 |
| iCHOv1 | 0.180 | 0.220 | 0.180 | 0.230 | 0.220 | 0.206 |
| iCHOv1_DG44 | 0.160 | 0.180 | 0.135 | 0.160 | 0.200 | 0.167 |
| iCN718 | 0.165 | 0.220 | 0.160 | 0.160 | 0.200 | 0.181 |
| iCN900 | 0.100 | 0.140 | 0.175 | 0.140 | 0.140 | 0.139 |
| iE2348C_1286 | 0.220 | 0.160 | 0.160 | 0.220 | 0.150 | 0.182 |
| iEC042_1314 | 0.160 | 0.145 | 0.120 | 0.120 | 0.140 | 0.137 |
| iEC1344_C | 0.060 | 0.060 | 0.060 | 0.060 | 0.075 | 0.063 |
| iEC1349_Crooks | 0.200 | 0.180 | 0.200 | 0.180 | 0.180 | 0.188 |
| iEC1356_Bl21DE3 | 0.120 | 0.120 | 0.120 | 0.075 | 0.100 | 0.107 |
| iEC1364_W | 0.260 | 0.220 | 0.260 | 0.220 | 0.200 | 0.232 |
| iEC1368_DH5a | 0.200 | 0.240 | 0.200 | 0.240 | 0.200 | 0.216 |
| iEC1372_W3110 | 0.240 | 0.200 | 0.220 | 0.180 | 0.170 | 0.202 |
| iEC55989_1330 | 0.120 | 0.100 | 0.120 | 0.120 | 0.080 | 0.108 |
| iECABU_c1320 | 0.145 | 0.120 | 0.160 | 0.180 | 0.140 | 0.149 |
| iECB_1328 | 0.080 | 0.110 | 0.120 | 0.080 | 0.120 | 0.102 |
| iECBD_1354 | 0.100 | 0.040 | 0.100 | 0.090 | 0.040 | 0.074 |
| iECD_1391 | 0.160 | 0.210 | 0.220 | 0.220 | 0.180 | 0.198 |
| iEcDH1_1363 | 0.180 | 0.200 | 0.220 | 0.180 | 0.225 | 0.201 |
| iECDH10B_1368 | 0.180 | 0.200 | 0.220 | 0.170 | 0.180 | 0.190 |
| iECDH1ME8569_1439 | 0.220 | 0.220 | 0.220 | 0.260 | 0.250 | 0.234 |
| iEcE24377_1341 | 0.200 | 0.205 | 0.240 | 0.240 | 0.200 | 0.217 |
| iECED1_1282 | 0.220 | 0.200 | 0.230 | 0.220 | 0.220 | 0.218 |
| iECH74115_1262 | 0.140 | 0.190 | 0.200 | 0.140 | 0.200 | 0.174 |
| iEcHS_1320 | 0.200 | 0.185 | 0.200 | 0.200 | 0.160 | 0.189 |
| iECIAI1_1343 | 0.080 | 0.050 | 0.100 | 0.100 | 0.080 | 0.082 |
| iECIAI39_1322 | 0.180 | 0.200 | 0.180 | 0.200 | 0.210 | 0.194 |
| iECNA114_1301 | 0.100 | 0.120 | 0.100 | 0.080 | 0.085 | 0.097 |
| iECO103_1326 | 0.220 | 0.195 | 0.200 | 0.200 | 0.180 | 0.199 |
| iECO111_1330 | 0.240 | 0.240 | 0.260 | 0.200 | 0.240 | 0.236 |

| GEMs | Top 50 (fold 0) | Top 50 (fold 1) | Top 50 (fold 2) | Top 50 (fold 3) | Top 50 (fold 4) | Top 50 (average) |
|---|---|---|---|---|---|---|
| iECO26_1355 | 0.140 | 0.140 | 0.155 | 0.140 | 0.180 | 0.151 |
| iECOK1_1307 | 0.140 | 0.180 | 0.180 | 0.215 | 0.180 | 0.179 |
| iEcolC_1368 | 0.200 | 0.240 | 0.240 | 0.220 | 0.240 | 0.228 |
| iECP_1309 | 0.060 | 0.080 | 0.100 | 0.090 | 0.060 | 0.078 |
| iECs_1301 | 0.245 | 0.180 | 0.200 | 0.220 | 0.180 | 0.205 |
| iECS88_1305 | 0.220 | 0.160 | 0.160 | 0.220 | 0.160 | 0.184 |
| iECSE_1348 | 0.160 | 0.120 | 0.120 | 0.160 | 0.165 | 0.145 |
| iECSF_1327 | 0.140 | 0.175 | 0.140 | 0.120 | 0.140 | 0.143 |
| iEcSMS35_1347 | 0.200 | 0.220 | 0.180 | 0.220 | 0.220 | 0.208 |
| iECSP_1301 | 0.140 | 0.165 | 0.160 | 0.160 | 0.140 | 0.153 |
| iECUMN_1333 | 0.140 | 0.120 | 0.140 | 0.170 | 0.140 | 0.142 |
| iECW_1372 | 0.200 | 0.180 | 0.200 | 0.180 | 0.200 | 0.192 |
| iEK1008 | 0.120 | 0.080 | 0.115 | 0.120 | 0.080 | 0.103 |
| iEKO11_1354 | 0.175 | 0.160 | 0.140 | 0.140 | 0.140 | 0.151 |
| iETEC_1333 | 0.260 | 0.260 | 0.240 | 0.240 | 0.280 | 0.256 |
| iG2583_1286 | 0.180 | 0.180 | 0.180 | 0.150 | 0.140 | 0.166 |
| iHN637 | 0.145 | 0.140 | 0.120 | 0.140 | 0.120 | 0.133 |
| iIS312 | 0.220 | 0.200 | 0.200 | 0.260 | 0.240 | 0.224 |
| iIS312_Amastigote | 0.080 | 0.120 | 0.120 | 0.070 | 0.120 | 0.102 |
| iIS312_Epimastigote | 0.160 | 0.125 | 0.140 | 0.100 | 0.160 | 0.137 |
| iIS312_Trypomastigote | 0.140 | 0.150 | 0.120 | 0.140 | 0.120 | 0.134 |
| iIT341 | 0.040 | 0.100 | 0.040 | 0.115 | 0.100 | 0.079 |
| iJB785 | 0.260 | 0.200 | 0.200 | 0.220 | 0.250 | 0.226 |
| iJN1463 | 0.080 | 0.100 | 0.150 | 0.100 | 0.140 | 0.114 |
| iJN678 | 0.165 | 0.200 | 0.160 | 0.220 | 0.220 | 0.193 |
| iJN746 | 0.120 | 0.125 | 0.160 | 0.120 | 0.180 | 0.141 |
| iJO1366 | 0.200 | 0.180 | 0.200 | 0.140 | 0.175 | 0.179 |
| iJR904 | 0.100 | 0.100 | 0.095 | 0.120 | 0.060 | 0.095 |
| iLB1027_lipid | 0.140 | 0.160 | 0.135 | 0.140 | 0.140 | 0.143 |
| iLF82_1304 | 0.060 | 0.060 | 0.120 | 0.100 | 0.120 | 0.092 |
| iLJ478 | 0.200 | 0.150 | 0.200 | 0.140 | 0.200 | 0.178 |
| iML1515 | 0.155 | 0.160 | 0.200 | 0.160 | 0.160 | 0.167 |
| iMM1415 | 0.200 | 0.200 | 0.180 | 0.200 | 0.180 | 0.192 |
| iMM904 | 0.220 | 0.160 | 0.220 | 0.160 | 0.160 | 0.184 |
| iND750 | 0.220 | 0.220 | 0.195 | 0.220 | 0.220 | 0.215 |
| iNF517 | 0.220 | 0.180 | 0.180 | 0.200 | 0.180 | 0.192 |
| iNJ661 | 0.140 | 0.180 | 0.160 | 0.160 | 0.190 | 0.166 |
| iNRG857_1313 | 0.160 | 0.225 | 0.200 | 0.220 | 0.160 | 0.193 |
| iPC815 | 0.065 | 0.080 | 0.120 | 0.080 | 0.120 | 0.093 |
| iRC1080 | 0.260 | 0.220 | 0.220 | 0.220 | 0.195 | 0.223 |
| iS_1188 | 0.105 | 0.140 | 0.140 | 0.140 | 0.120 | 0.129 |
| iSB619 | 0.220 | 0.205 | 0.200 | 0.160 | 0.200 | 0.197 |
| iSbBS512_1146 | 0.140 | 0.140 | 0.140 | 0.125 | 0.160 | 0.141 |
| iSBO_1134 | 0.120 | 0.140 | 0.080 | 0.075 | 0.120 | 0.107 |
| iSDY_1059 | 0.120 | 0.080 | 0.120 | 0.120 | 0.145 | 0.117 |

| GEMs | Top 50 (fold 0) | Top 50 (fold 1) | Top 50 (fold 2) | Top 50 (fold 3) | Top 50 (fold 4) | Top 50 (average) |
|---|---|---|---|---|---|---|
| iSF_1195 | 0.120 | 0.160 | 0.165 | 0.120 | 0.160 | 0.145 |
| iSFV_1184 | 0.160 | 0.140 | 0.160 | 0.200 | 0.160 | 0.164 |
| iSFxv_1172 | 0.135 | 0.140 | 0.180 | 0.140 | 0.140 | 0.147 |
| iSSON_1240 | 0.105 | 0.100 | 0.080 | 0.060 | 0.080 | 0.085 |
| iSynCJ816 | 0.140 | 0.140 | 0.120 | 0.100 | 0.115 | 0.123 |
| iUMN146_1321 | 0.140 | 0.160 | 0.140 | 0.180 | 0.195 | 0.163 |
| iUMNK88_1353 | 0.160 | 0.180 | 0.140 | 0.200 | 0.200 | 0.176 |
| iUTI89_1310 | 0.220 | 0.250 | 0.200 | 0.200 | 0.240 | 0.222 |
| iWFL_1372 | 0.225 | 0.200 | 0.200 | 0.200 | 0.220 | 0.209 |
| iY75_1357 | 0.200 | 0.185 | 0.180 | 0.200 | 0.200 | 0.193 |
| iYL1228 | 0.120 | 0.080 | 0.115 | 0.060 | 0.120 | 0.099 |
| iYO844 | 0.140 | 0.155 | 0.100 | 0.140 | 0.140 | 0.135 |
| iYS1720 | 0.155 | 0.160 | 0.180 | 0.140 | 0.160 | 0.159 |
| iYS854 | 0.240 | 0.180 | 0.200 | 0.240 | 0.200 | 0.212 |
| iZ_1308 | 0.140 | 0.120 | 0.140 | 0.140 | 0.170 | 0.142 |
| RECON1 | 0.200 | 0.155 | 0.220 | 0.160 | 0.200 | 0.187 |
| Recon3D | 0.120 | 0.120 | 0.140 | 0.140 | 0.090 | 0.122 |
| STM_v1_0 | 0.150 | 0.160 | 0.160 | 0.160 | 0.140 | 0.154 |

| GEMs | Top 100 (fold 0) | Top 100 (fold 1) | Top 100 (fold 2) | Top 100 (fold 3) | Top 100 (fold 4) | Top 100 (average) |
|---|---|---|---|---|---|---|
| e_coli_core | 0.045 | 0.080 | 0.090 | 0.090 | 0.030 | 0.067 |
| iAB_RBC_283 | 0.190 | 0.240 | 0.230 | 0.200 | 0.185 | 0.209 |
| iAF1260 | 0.070 | 0.100 | 0.070 | 0.065 | 0.030 | 0.067 |
| iAF1260b | 0.140 | 0.070 | 0.090 | 0.135 | 0.100 | 0.107 |
| iAF692 | 0.165 | 0.210 | 0.220 | 0.170 | 0.180 | 0.189 |
| iAF987 | 0.080 | 0.060 | 0.090 | 0.130 | 0.130 | 0.098 |
| iAM_Pb448 | 0.160 | 0.160 | 0.135 | 0.140 | 0.130 | 0.145 |
| iAM_Pc455 | 0.145 | 0.100 | 0.120 | 0.130 | 0.120 | 0.123 |
| iAM_Pf480 | 0.110 | 0.105 | 0.170 | 0.170 | 0.130 | 0.137 |
| iAM_Pk459 | 0.060 | 0.100 | 0.080 | 0.040 | 0.040 | 0.064 |
| iAM_Pv461 | 0.100 | 0.070 | 0.070 | 0.110 | 0.050 | 0.080 |
| iAPECO1_1312 | 0.090 | 0.130 | 0.140 | 0.110 | 0.155 | 0.125 |
| iAT_PLT_636 | 0.140 | 0.160 | 0.160 | 0.200 | 0.210 | 0.174 |
| iB21_1397 | 0.180 | 0.160 | 0.160 | 0.180 | 0.180 | 0.172 |
| iBWG_1329 | 0.150 | 0.150 | 0.170 | 0.190 | 0.200 | 0.172 |
| ic_1306 | 0.140 | 0.150 | 0.150 | 0.210 | 0.210 | 0.172 |
| iCHOv1 | 0.245 | 0.210 | 0.210 | 0.210 | 0.260 | 0.227 |
| iCHOv1_DG44 | 0.210 | 0.150 | 0.210 | 0.180 | 0.180 | 0.186 |
| iCN718 | 0.150 | 0.125 | 0.090 | 0.130 | 0.090 | 0.117 |
| iCN900 | 0.180 | 0.245 | 0.180 | 0.180 | 0.240 | 0.205 |
| iE2348C_1286 | 0.130 | 0.070 | 0.130 | 0.100 | 0.080 | 0.102 |
| iEC042_1314 | 0.140 | 0.080 | 0.095 | 0.120 | 0.140 | 0.115 |
| iEC1344_C | 0.200 | 0.250 | 0.230 | 0.260 | 0.200 | 0.228 |
| iEC1349_Crooks | 0.140 | 0.210 | 0.200 | 0.180 | 0.165 | 0.179 |
| iEC1356_Bl21DE3 | 0.190 | 0.175 | 0.210 | 0.180 | 0.230 | 0.197 |
| iEC1364_W | 0.090 | 0.070 | 0.025 | 0.050 | 0.080 | 0.063 |
| iEC1368_DH5a | 0.180 | 0.135 | 0.160 | 0.170 | 0.160 | 0.161 |
| iEC1372_W3110 | 0.240 | 0.210 | 0.275 | 0.210 | 0.270 | 0.241 |
| iEC55989_1330 | 0.210 | 0.190 | 0.190 | 0.240 | 0.250 | 0.216 |
| iECABU_c1320 | 0.040 | 0.080 | 0.110 | 0.110 | 0.050 | 0.078 |
| iECB_1328 | 0.230 | 0.230 | 0.230 | 0.250 | 0.280 | 0.244 |
| iECBD_1354 | 0.200 | 0.240 | 0.170 | 0.230 | 0.200 | 0.208 |
| iECD_1391 | 0.090 | 0.060 | 0.090 | 0.120 | 0.050 | 0.082 |
| iEcDH1_1363 | 0.170 | 0.180 | 0.190 | 0.240 | 0.220 | 0.200 |
| iECDH10B_1368 | 0.100 | 0.120 | 0.100 | 0.060 | 0.075 | 0.091 |
| iECDH1ME8569_1439 | 0.240 | 0.260 | 0.250 | 0.210 | 0.230 | 0.238 |
| iEcE24377_1341 | 0.220 | 0.200 | 0.225 | 0.190 | 0.250 | 0.217 |
| iECED1_1282 | 0.030 | 0.090 | 0.070 | 0.070 | 0.015 | 0.055 |
| iECH74115_1262 | 0.235 | 0.220 | 0.220 | 0.220 | 0.190 | 0.217 |
| iEcHS_1320 | 0.160 | 0.120 | 0.130 | 0.180 | 0.180 | 0.154 |
| iECIAI1_1343 | 0.100 | 0.060 | 0.085 | 0.060 | 0.110 | 0.083 |
| iECIAI39_1322 | 0.130 | 0.070 | 0.110 | 0.070 | 0.110 | 0.098 |
| iECNA114_1301 | 0.260 | 0.190 | 0.240 | 0.220 | 0.240 | 0.230 |
| iECO103_1326 | 0.100 | 0.100 | 0.085 | 0.160 | 0.160 | 0.121 |
| iECO111_1330 | 0.140 | 0.120 | 0.150 | 0.120 | 0.090 | 0.124 |

| GEMs | Top 100 (fold 0) | Top 100 (fold 1) | Top 100 (fold 2) | Top 100 (fold 3) | Top 100 (fold 4) | Top 100 (average) |
|---|---|---|---|---|---|---|
| iECO26_1355 | 0.190 | 0.230 | 0.170 | 0.190 | 0.175 | 0.191 |
| iECOK1_1307 | 0.080 | 0.140 | 0.140 | 0.075 | 0.130 | 0.113 |
| iEcolC_1368 | 0.130 | 0.090 | 0.090 | 0.090 | 0.055 | 0.091 |
| iECP_1309 | 0.170 | 0.175 | 0.170 | 0.190 | 0.180 | 0.177 |
| iECs_1301 | 0.220 | 0.220 | 0.250 | 0.240 | 0.200 | 0.226 |
| iECS88_1305 | 0.220 | 0.220 | 0.220 | 0.220 | 0.210 | 0.218 |
| iECSE_1348 | 0.190 | 0.245 | 0.240 | 0.200 | 0.230 | 0.221 |
| iECSF_1327 | 0.210 | 0.220 | 0.180 | 0.210 | 0.210 | 0.206 |
| iEcSMS35_1347 | 0.110 | 0.090 | 0.090 | 0.100 | 0.090 | 0.096 |
| iECSP_1301 | 0.250 | 0.245 | 0.200 | 0.220 | 0.200 | 0.223 |
| iECUMN_1333 | 0.100 | 0.120 | 0.140 | 0.110 | 0.130 | 0.120 |
| iECW_1372 | 0.110 | 0.070 | 0.130 | 0.100 | 0.130 | 0.108 |
| iEK1008 | 0.130 | 0.160 | 0.130 | 0.180 | 0.110 | 0.142 |
| iEKO11_1354 | 0.190 | 0.210 | 0.160 | 0.160 | 0.150 | 0.174 |
| iETEC_1333 | 0.150 | 0.195 | 0.190 | 0.190 | 0.190 | 0.183 |
| iG2583_1286 | 0.210 | 0.210 | 0.210 | 0.180 | 0.160 | 0.194 |
| iHN637 | 0.150 | 0.200 | 0.180 | 0.150 | 0.150 | 0.166 |
| iIS312 | 0.100 | 0.080 | 0.100 | 0.140 | 0.080 | 0.100 |
| iIS312_Amastigote | 0.090 | 0.120 | 0.100 | 0.120 | 0.100 | 0.106 |
| iIS312_Epimastigote | 0.150 | 0.150 | 0.150 | 0.150 | 0.200 | 0.160 |
| iIS312_Trypomastigote | 0.070 | 0.110 | 0.060 | 0.070 | 0.110 | 0.084 |
| iIT341 | 0.150 | 0.140 | 0.130 | 0.090 | 0.130 | 0.128 |
| iJB785 | 0.140 | 0.160 | 0.130 | 0.130 | 0.190 | 0.150 |
| iJN1463 | 0.080 | 0.080 | 0.140 | 0.110 | 0.110 | 0.104 |
| iJN678 | 0.190 | 0.215 | 0.190 | 0.210 | 0.160 | 0.193 |
| iJN746 | 0.030 | 0.090 | 0.100 | 0.035 | 0.050 | 0.061 |
| iJO1366 | 0.175 | 0.230 | 0.200 | 0.190 | 0.200 | 0.199 |
| iJR904 | 0.220 | 0.210 | 0.230 | 0.180 | 0.250 | 0.218 |
| iLB1027_lipid | 0.090 | 0.110 | 0.090 | 0.110 | 0.150 | 0.110 |
| iLF82_1304 | 0.170 | 0.170 | 0.160 | 0.160 | 0.200 | 0.172 |
| iLJ478 | 0.090 | 0.040 | 0.040 | 0.100 | 0.080 | 0.070 |
| iML1515 | 0.200 | 0.220 | 0.190 | 0.200 | 0.230 | 0.208 |
| iMM1415 | 0.080 | 0.120 | 0.050 | 0.100 | 0.065 | 0.083 |
| iMM904 | 0.090 | 0.070 | 0.090 | 0.130 | 0.110 | 0.098 |
| iND750 | 0.140 | 0.140 | 0.105 | 0.160 | 0.140 | 0.137 |
| iNF517 | 0.135 | 0.140 | 0.110 | 0.150 | 0.120 | 0.131 |
| iNJ661 | 0.090 | 0.140 | 0.100 | 0.160 | 0.120 | 0.122 |
| iNRG857_1313 | 0.110 | 0.110 | 0.080 | 0.110 | 0.080 | 0.098 |
| iPC815 | 0.240 | 0.240 | 0.240 | 0.230 | 0.240 | 0.238 |
| iRC1080 | 0.150 | 0.140 | 0.210 | 0.210 | 0.140 | 0.170 |
| iS_1188 | 0.130 | 0.160 | 0.170 | 0.160 | 0.135 | 0.151 |
| iSB619 | 0.070 | 0.120 | 0.070 | 0.070 | 0.120 | 0.090 |
| iSbBS512_1146 | 0.140 | 0.130 | 0.130 | 0.150 | 0.160 | 0.142 |
| iSBO_1134 | 0.120 | 0.110 | 0.170 | 0.160 | 0.170 | 0.146 |
| iSDY_1059 | 0.075 | 0.070 | 0.070 | 0.030 | 0.070 | 0.063 |

| GEMs | Top 100 (fold 0) | Top 100 (fold 1) | Top 100 (fold 2) | Top 100 (fold 3) | Top 100 (fold 4) | Top 100 (average) |
|---|---|---|---|---|---|---|
| iSF_1195 | 0.070 | 0.120 | 0.100 | 0.140 | 0.080 | 0.102 |
| iSFV_1184 | 0.110 | 0.050 | 0.090 | 0.080 | 0.105 | 0.087 |
| iSFxv_1172 | 0.170 | 0.170 | 0.220 | 0.190 | 0.230 | 0.196 |
| iSSON_1240 | 0.180 | 0.160 | 0.160 | 0.190 | 0.130 | 0.164 |
| iSynCJ816 | 0.170 | 0.170 | 0.160 | 0.195 | 0.170 | 0.173 |
| iUMN146_1321 | 0.175 | 0.210 | 0.240 | 0.210 | 0.190 | 0.205 |
| iUMNK88_1353 | 0.110 | 0.140 | 0.140 | 0.090 | 0.110 | 0.118 |
| iUTI89_1310 | 0.240 | 0.200 | 0.250 | 0.215 | 0.200 | 0.221 |
| iWFL_1372 | 0.150 | 0.170 | 0.200 | 0.200 | 0.210 | 0.186 |
| iY75_1357 | 0.220 | 0.210 | 0.180 | 0.230 | 0.180 | 0.204 |
| iYL1228 | 0.220 | 0.190 | 0.220 | 0.190 | 0.240 | 0.212 |
| iYO844 | 0.190 | 0.130 | 0.145 | 0.170 | 0.140 | 0.155 |
| iYS1720 | 0.230 | 0.235 | 0.250 | 0.200 | 0.220 | 0.227 |
| iYS854 | 0.110 | 0.125 | 0.080 | 0.060 | 0.060 | 0.087 |
| iZ_1308 | 0.155 | 0.200 | 0.170 | 0.220 | 0.210 | 0.191 |
| RECON1 | 0.110 | 0.130 | 0.110 | 0.170 | 0.150 | 0.134 |
| Recon3D | 0.160 | 0.190 | 0.190 | 0.160 | 0.160 | 0.172 |
| STM_v1_0 | 0.180 | 0.190 | 0.185 | 0.150 | 0.180 | 0.177 |

| GEMs | Top N (fold 0) | Top N (fold 1) | Top N (fold 2) | Top N (fold 3) | Top N (fold 4) | Top N (average) |
|---|---|---|---|---|---|---|
| e_coli_core | 0.085 | 0.050 | 0.067 | 0.054 | 0.094 | 0.070 |
| iAB_RBC_283 | 0.044 | 0.075 | 0.028 | 0.065 | 0.088 | 0.060 |
| iAF1260 | 0.103 | 0.079 | 0.140 | 0.137 | 0.101 | 0.112 |
| iAF1260b | 0.153 | 0.159 | 0.111 | 0.090 | 0.122 | 0.127 |
| iAF692 | 0.027 | 0.039 | 0.066 | 0.067 | 0.066 | 0.053 |
| iAF987 | 0.030 | 0.084 | 0.052 | 0.070 | 0.064 | 0.060 |
| iAM_Pb448 | 0.078 | 0.076 | 0.088 | 0.067 | 0.101 | 0.082 |
| iAM_Pc455 | 0.066 | 0.125 | 0.117 | 0.076 | 0.111 | 0.099 |
| iAM_Pf480 | 0.082 | 0.057 | 0.108 | 0.087 | 0.076 | 0.082 |
| iAM_Pk459 | 0.145 | 0.104 | 0.142 | 0.106 | 0.158 | 0.131 |
| iAM_Pv461 | 0.159 | 0.144 | 0.173 | 0.145 | 0.204 | 0.165 |
| iAPECO1_1312 | 0.078 | 0.108 | 0.037 | 0.097 | 0.060 | 0.076 |
| iAT_PLT_636 | 0.126 | 0.082 | 0.069 | 0.064 | 0.109 | 0.090 |
| iB21_1397 | 0.128 | 0.126 | 0.161 | 0.200 | 0.190 | 0.161 |
| iBWG_1329 | 0.105 | 0.110 | 0.100 | 0.102 | 0.088 | 0.101 |
| ic_1306 | 0.111 | 0.072 | 0.120 | 0.103 | 0.134 | 0.108 |
| iCHOv1 | 0.199 | 0.188 | 0.231 | 0.258 | 0.224 | 0.220 |
| iCHOv1_DG44 | 0.079 | 0.061 | 0.046 | 0.113 | 0.086 | 0.077 |
| iCN718 | 0.048 | 0.074 | 0.079 | 0.071 | 0.098 | 0.074 |
| iCN900 | 0.149 | 0.206 | 0.179 | 0.213 | 0.143 | 0.178 |
| iE2348C_1286 | 0.126 | 0.104 | 0.142 | 0.109 | 0.104 | 0.117 |
| iEC042_1314 | 0.070 | 0.059 | 0.062 | 0.095 | 0.079 | 0.073 |
| iEC1344_C | 0.133 | 0.121 | 0.162 | 0.123 | 0.151 | 0.138 |
| iEC1349_Crooks | 0.111 | 0.148 | 0.177 | 0.112 | 0.172 | 0.144 |
| iEC1356_Bl21DE3 | 0.162 | 0.091 | 0.113 | 0.085 | 0.159 | 0.122 |
| iEC1364_W | 0.173 | 0.213 | 0.147 | 0.207 | 0.185 | 0.185 |
| iEC1368_DH5a | 0.132 | 0.133 | 0.137 | 0.175 | 0.193 | 0.154 |
| iEC1372_W3110 | 0.161 | 0.140 | 0.125 | 0.184 | 0.140 | 0.150 |
| iEC55989_1330 | 0.201 | 0.156 | 0.156 | 0.222 | 0.180 | 0.183 |
| iECABU_c1320 | 0.148 | 0.119 | 0.162 | 0.169 | 0.162 | 0.152 |
| iECB_1328 | 0.142 | 0.142 | 0.132 | 0.170 | 0.174 | 0.152 |
| iECBD_1354 | 0.095 | 0.064 | 0.048 | 0.070 | 0.108 | 0.077 |
| iECD_1391 | 0.075 | 0.035 | 0.068 | 0.035 | 0.082 | 0.059 |
| iEcDH1_1363 | 0.149 | 0.202 | 0.136 | 0.197 | 0.161 | 0.169 |
| iECDH10B_1368 | 0.104 | 0.131 | 0.113 | 0.139 | 0.153 | 0.128 |
| iECDH1ME8569_1439 | 0.125 | 0.077 | 0.119 | 0.106 | 0.138 | 0.113 |
| iEcE24377_1341 | 0.158 | 0.142 | 0.195 | 0.174 | 0.196 | 0.173 |
| iECED1_1282 | 0.189 | 0.248 | 0.210 | 0.242 | 0.186 | 0.215 |
| iECH74115_1262 | 0.136 | 0.195 | 0.168 | 0.174 | 0.152 | 0.165 |
| iEcHS_1320 | 0.192 | 0.203 | 0.223 | 0.192 | 0.205 | 0.203 |
| iECIAI1_1343 | 0.214 | 0.162 | 0.230 | 0.154 | 0.200 | 0.192 |
| iECIAI39_1322 | 0.165 | 0.149 | 0.128 | 0.107 | 0.171 | 0.144 |
| iECNA114_1301 | 0.177 | 0.176 | 0.190 | 0.172 | 0.180 | 0.179 |
| iECO103_1326 | 0.146 | 0.133 | 0.134 | 0.077 | 0.090 | 0.116 |
| iECO111_1330 | 0.099 | 0.130 | 0.081 | 0.073 | 0.072 | 0.091 |

| GEMs | Top N (fold 0) | Top N (fold 1) | Top N (fold 2) | Top N (fold 3) | Top N (fold 4) | Top N (average) |
|---|---|---|---|---|---|---|
| iECO26_1355 | 0.145 | 0.123 | 0.182 | 0.128 | 0.177 | 0.151 |
| iECOK1_1307 | 0.120 | 0.137 | 0.174 | 0.157 | 0.117 | 0.141 |
| iEcolC_1368 | 0.081 | 0.095 | 0.112 | 0.108 | 0.109 | 0.101 |
| iECP_1309 | 0.124 | 0.125 | 0.134 | 0.075 | 0.117 | 0.115 |
| iECs_1301 | 0.065 | 0.113 | 0.111 | 0.069 | 0.102 | 0.092 |
| iECS88_1305 | 0.101 | 0.125 | 0.117 | 0.104 | 0.118 | 0.113 |
| iECSE_1348 | 0.118 | 0.136 | 0.098 | 0.160 | 0.163 | 0.135 |
| iECSF_1327 | 0.143 | 0.111 | 0.123 | 0.139 | 0.139 | 0.131 |
| iEcSMS35_1347 | 0.149 | 0.152 | 0.126 | 0.166 | 0.142 | 0.147 |
| iECSP_1301 | 0.158 | 0.164 | 0.197 | 0.140 | 0.161 | 0.164 |
| iECUMN_1333 | 0.146 | 0.153 | 0.164 | 0.153 | 0.139 | 0.151 |
| iECW_1372 | 0.164 | 0.148 | 0.181 | 0.209 | 0.193 | 0.179 |
| iEK1008 | 0.142 | 0.156 | 0.095 | 0.130 | 0.152 | 0.135 |
| iEKO11_1354 | 0.115 | 0.072 | 0.088 | 0.136 | 0.119 | 0.106 |
| iETEC_1333 | 0.077 | 0.074 | 0.083 | 0.083 | 0.098 | 0.083 |
| iG2583_1286 | 0.191 | 0.176 | 0.142 | 0.134 | 0.127 | 0.154 |
| iHN637 | 0.054 | 0.084 | 0.056 | 0.080 | 0.101 | 0.075 |
| iIS312 | 0.059 | 0.084 | 0.019 | 0.028 | 0.080 | 0.054 |
| iIS312_Amastigote | 0.162 | 0.133 | 0.168 | 0.171 | 0.146 | 0.156 |
| iIS312_Epimastigote | 0.097 | 0.134 | 0.066 | 0.138 | 0.090 | 0.105 |
| iIS312_Trypomastigote | 0.097 | 0.057 | 0.066 | 0.088 | 0.082 | 0.078 |
| iIT341 | 0.122 | 0.109 | 0.072 | 0.100 | 0.147 | 0.110 |
| iJB785 | 0.052 | 0.098 | 0.104 | 0.042 | 0.029 | 0.065 |
| iJN1463 | 0.080 | 0.118 | 0.134 | 0.098 | 0.060 | 0.098 |
| iJN678 | 0.118 | 0.119 | 0.095 | 0.168 | 0.165 | 0.133 |
| iJN746 | 0.017 | 0.018 | 0.086 | 0.060 | 0.079 | 0.052 |
| iJO1366 | 0.154 | 0.138 | 0.165 | 0.189 | 0.169 | 0.163 |
| iJR904 | 0.079 | 0.052 | 0.030 | 0.073 | 0.071 | 0.061 |
| iLB1027_lipid | 0.080 | 0.025 | 0.099 | 0.073 | 0.033 | 0.062 |
| iLF82_1304 | 0.194 | 0.207 | 0.224 | 0.159 | 0.161 | 0.189 |
| iLJ478 | 0.216 | 0.180 | 0.203 | 0.241 | 0.220 | 0.212 |
| iML1515 | 0.149 | 0.105 | 0.122 | 0.160 | 0.089 | 0.125 |
| iMM1415 | 0.224 | 0.163 | 0.172 | 0.194 | 0.172 | 0.185 |
| iMM904 | 0.159 | 0.135 | 0.162 | 0.123 | 0.121 | 0.140 |
| iND750 | 0.152 | 0.156 | 0.121 | 0.184 | 0.177 | 0.158 |
| iNF517 | 0.227 | 0.180 | 0.192 | 0.164 | 0.192 | 0.191 |
| iNJ661 | 0.172 | 0.208 | 0.165 | 0.168 | 0.197 | 0.182 |
| iNRG857_1313 | 0.170 | 0.188 | 0.231 | 0.201 | 0.195 | 0.197 |
| iPC815 | 0.168 | 0.141 | 0.115 | 0.120 | 0.111 | 0.131 |
| iRC1080 | 0.242 | 0.195 | 0.191 | 0.163 | 0.219 | 0.202 |
| iS_1188 | 0.183 | 0.138 | 0.174 | 0.150 | 0.155 | 0.160 |
| iSB619 | 0.202 | 0.183 | 0.149 | 0.194 | 0.192 | 0.184 |
| iSbBS512_1146 | 0.225 | 0.224 | 0.166 | 0.184 | 0.231 | 0.206 |
| iSBO_1134 | 0.163 | 0.113 | 0.103 | 0.148 | 0.148 | 0.135 |
| iSDY_1059 | 0.101 | 0.055 | 0.113 | 0.072 | 0.119 | 0.092 |

| GEMs | Top N (fold 0) | Top N (fold 1) | Top N (fold 2) | Top N (fold 3) | Top N (fold 4) | Top N (average) |
|---|---|---|---|---|---|---|
| iSF_1195 | 0.139 | 0.170 | 0.137 | 0.121 | 0.163 | 0.146 |
| iSFV_1184 | 0.187 | 0.161 | 0.213 | 0.188 | 0.191 | 0.188 |
| iSFxv_1172 | 0.103 | 0.107 | 0.162 | 0.136 | 0.157 | 0.133 |
| iSSON_1240 | 0.218 | 0.222 | 0.185 | 0.218 | 0.182 | 0.205 |
| iSynCJ816 | 0.206 | 0.180 | 0.163 | 0.223 | 0.213 | 0.197 |
| iUMN146_1321 | 0.094 | 0.155 | 0.129 | 0.080 | 0.122 | 0.116 |
| iUMNK88_1353 | 0.137 | 0.208 | 0.197 | 0.194 | 0.134 | 0.174 |
| iUTI89_1310 | 0.124 | 0.090 | 0.092 | 0.117 | 0.147 | 0.114 |
| iWFL_1372 | 0.153 | 0.126 | 0.115 | 0.100 | 0.141 | 0.127 |
| iY75_1357 | 0.113 | 0.065 | 0.062 | 0.108 | 0.102 | 0.090 |
| iYL1228 | 0.064 | 0.043 | 0.074 | 0.043 | 0.081 | 0.061 |
| iYO844 | 0.114 | 0.129 | 0.113 | 0.078 | 0.146 | 0.116 |
| iYS1720 | 0.043 | 0.054 | 0.106 | 0.064 | 0.078 | 0.069 |
| iYS854 | 0.202 | 0.179 | 0.185 | 0.151 | 0.223 | 0.188 |
| iZ_1308 | 0.187 | 0.192 | 0.139 | 0.160 | 0.122 | 0.160 |
| RECON1 | 0.223 | 0.237 | 0.231 | 0.193 | 0.171 | 0.211 |
| Recon3D | 0.221 | 0.207 | 0.200 | 0.161 | 0.161 | 0.190 |
| STM_v1_0 | 0.049 | 0.089 | 0.064 | 0.018 | 0.040 | 0.052 |
